# A Survey of Underwater Acoustic Data Classification Methods Using Deep Learning for Shoreline Surveillance

**DOI:** 10.3390/s22062181

**Published:** 2022-03-11

**Authors:** Lucas C. F. Domingos, Paulo E. Santos, Phillip S. M. Skelton, Russell S. A. Brinkworth, Karl Sammut

**Affiliations:** 1 Department of Electrical and Electronics Engineering, Centro Universitário FEI, Sao Bernardo do Campo 09850-901, SP, Brazil; paulo.santos@flinders.edu.au; 2 Department of Computer Vision, Instituto de Pesquisas Eldorado, Campinas 13083-898, SP, Brazil; 3 Centre for Defence Engineering Research and Training, College of Science and Engineering, Flinders University, Tonsley, SA 5042, Australia; phillip.skelton@flinders.edu.au (P.S.M.S.); russell.brinkworth@flinders.edu.au (R.S.A.B.); karl.sammut@flinders.edu.au (K.S.)

**Keywords:** deep convolutional neural networks, underwater acoustics, objects’ classification

## Abstract

This paper presents a comprehensive overview of current deep-learning methods for automatic object classification of underwater sonar data for shoreline surveillance, concentrating mostly on the classification of vessels from passive sonar data and the identification of objects of interest from active sonar (such as minelike objects, human figures or debris of wrecked ships). Not only is the contribution of this work to provide a systematic description of the state of the art of this field, but also to identify five main ingredients in its current development: the application of deep-learning methods using convolutional layers alone; deep-learning methods that apply biologically inspired feature-extraction filters as a preprocessing step; classification of data from frequency and time–frequency analysis; methods using machine learning to extract features from original signals; and transfer learning methods. This paper also describes some of the most important datasets cited in the literature and discusses data-augmentation techniques. The latter are used for coping with the scarcity of annotated sonar datasets from real maritime missions.

## 1. Introduction

The importance of developing accurate automatic object classification methods for underwater sensor data in general, and Sound Navigation and Ranging (sonar) data in particular, is directly related to the variety of potential applications depending on them. Examples of marine classification tasks include the inspection of underwater structures for the offshore industry [1,2], the identification of underwater archaeological remains [3], the surveillance of shorelines [4], counting and classifying the behaviour of marine life for biological research [5] and the identification of vessels [6] to cite a few. Acoustic signals in the sea also mean environmental pollution affecting marine life [7,8,9] and humans where ships are nearby port areas [10,11,12]. However, the present paper is only concerned with object detection and classification from sonar data. Traditionally, human sonar operators have been primarily responsible for this task. When it comes to computer analysis, early work in this field predominantly comprised time–frequency analyses, such as the use of Fourier transforms to temporal data segments [13]. In contrast, most recent work is based on the application of deep-learning methods to accomplish this task in an automatic way [14,15,16]. Due to the rapid development of this field in recent years, a systematic review of deep-learning methods applied to the classification of underwater acoustic data is a timely and relevant task.

This paper presents an up-to-date literature review of current deep-learning methods for automatic object classification using underwater sonar data for the surveillance of littoral waters. Therefore, this paper will concentrate mostly, but not solely, on the classification of vessels from passive sonar data and the identification of objects of interest from active sonar (such as minelike objects, human figures or debris from wrecked ships). In general terms, the contribution of this work is to provide a systematic description of the state of the art of this field, highlighting important methods, strategies and published results in the classification of acoustic underwater signals by current machine-learning methods. In particular, this review identifies five main strategies cited in the current literature on the subject: the application of Deep Learning (DL) methods using convolutional layers alone (Section 3.1); DL methods that apply biologically inspired feature extraction filters as a preprocessing step (Section 3.2); classification of data from frequency and time–frequency analysis (Section 3.3); methods using machine learning to extract features from original signals (Section 3.4); and transfer learning methods, that aim to use pretrained models fine-tuned with small sonar datasets (Section 3.5). This paper also describes the various datasets cited in the literature (Section 4), and discusses the desired features that a dataset should have to push forward the research boundaries of this field. There is a clear scarcity of publicly available, labelled datasets of underwater acoustic signals, preventing reproducibility and repeatability of the results, greatly hindering the development of this field (in contrast with the fast progress of image classification in the past few years [17]). In order to cope with this issue, and also with the complexity in obtaining sonar data from real maritime missions, besides the use of transfer learning, various data-augmentation techniques are described in the literature (summarised in Section 4.1). Early techniques and datasets used in this task have been summarised previously in [14,15,16,18], and the present survey contributes to this group by collating the main ingredients needed to accelerate the development of this area, namely, an up-to-date account of the current methods, existing datasets, and a summary of techniques commonly used for solving the small sample size problem: data augmentation and transfer learning.

The next section introduces the background knowledge on the physics of underwater acoustics (Section 2.1), classic sound processing methods (Section 2.3), and Deep Neural Networks (Section 2.4), that constitute the context in which research on the classification of underwater acoustic data is developed.

## 2. Background Knowledge

This section presents a summary of underwater acoustics, classic signal processing methods and general deep-learning algorithms. These constitute the background knowledge needed to understand and analyse the current methods for the autonomous classification of underwater sonar data in the maritime domain.

### 2.1. Underwater Acoustics

The acoustic signal produced by a vessel moving in the sea is mainly composed of a broadband component (with a continuous spectrum), that is generated by the propeller and its hydrodynamic interactions; and a narrow band component (whose spectrum consists of line components at discrete frequencies), owing to the propulsion system and other mechanical parts [19]. The automatic classification of this type of signal is a challenging task, as the signal is also dependent on the vessel’s speed, the age and state of the propulsion system, the highly variable background noise and the diversity of sound propagation mechanisms in the ocean. The latter aspect is also a source of complexity in active sonar applications. As identified in Section 3 below, the characteristics of sound propagation underwater, however, are largely ignored in most literature concerning machine-learning-based underwater acoustic signal classifiers, even though it is essential for interpreting the accuracy of the classification results. In order to provide an appropriate context for this issue, this section presents a brief summary of underwater acoustics, mainly based on [20,21,22].

Sound propagation in the ocean is dependent on properties of the water column (temperature, salinity and pressure) and effects related to the ocean floor (scattering and reverberation). For a temperature *T* (measured in Celsius), a depth below the surface *z* (in meters) and salinity *S* (parts per thousand), the sound speed (c) in the ocean can be represented by the following equation [21]:

(1) $$\begin{array}{c} c=1449.2+4.6T+0.055T^{2}+1.39\left(S-35\right)+0.016z. \end{array}$$

Equation (1) is one possible way of defining the dependency of the speed of sound with oceanographic variables, other formulations are presented in [23].

Sound rays respect Snell’s law of refraction, which expresses that the rate between the cosine of the ray angle θ(z), with respect to the horizontal plane, at local sound speed c(z) and depth z, is constant:

(2) $$\begin{array}{c} \frac{cos\theta (z)}{c(z)}=constant. \end{array}$$

Therefore, a negative sound–speed gradient (e.g., in a thermocline) causes sound rays to bend downwards; the opposite happens with a positive gradient (e.g., in deep ocean where changes in pressure are greater than changes in temperature). In other words, sound bends toward regions of low sound speed [20]. This implies that distinct sound profiles should be considered for warmer and colder geographic regions, time periods of the day or seasons of the year. Ocean volume also causes attenuation of the sound signal, which is directly proportional to the acoustic frequency.

In more general terms, the influences of oceanographic properties on the sound propagation paths can be classified into three basic classes: *very short range*, *deep water*, and *shallow water propagation* [20]. Very short range propagation includes the direct path and the surface reflected path, the former refers to sound waves that travel without interacting with the sea surface or bottom; the latter refers to sound waves that are reflected by the air–sea interface. The interference of these two paths creates the so-called Lloyd mirror pattern [24], which makes object detection and classification in shallow waters (such as in a harbour or port) a challenging task [25]. Deep water (or long range) propagation paths can mostly be characterised by Snell’s law of refraction, apart from the *bottom bounce* effect, in which sound rays are reflected by the ocean floor in a process that is dependent on the signal frequency and that is also sensitive to seabed characteristics. In shallow waters (depths up to a few hundred meters from the surface to the ocean bottom), the effects of surface reflection, bottom bounce and distinct temperature regimes in distinct seasons have to be taken into account. During the summer period, following Snell’s law, sound rays bend more toward the bottom than during the winder months, this implies that the bottom bounce effect is more prominent during the hotter periods of the year, and therefore, the sound propagation in shallow waters has higher losses in the summer than during the winter. The rough winter surface conditions also have to be considered, as they generate large scattering losses at high frequencies, since more energy is needed to maintain the mechanical vibrations in these situations [22].

Scattering, due to rough boundaries or small obstacles, is another process that causes loss in the acoustic signal. In contrast to reflection, scattering happens with wavelengths at the order of the obstacles, causing parts of the acoustic field to be randomised [26]. Scattering due to rough surfaces cause a frequency-dependent attenuation of the acoustic field, whereas volume scattering (usually due to near-surface bubbles, bubble clouds or biological obstacles, such as air-filled swim bladders and zooplankton) decreases with depth and presents variations at distinct periods of the day [20].

Classical signal processing methods, such as cepstral analysis [13], are able to attenuate some of the effects of reflection interference and scattering losses, but only at short ranges with a high signal-to-noise ratio [25]. Nevertheless, these effects represent some of the main challenges for sonar systems (briefly introduced below), and they should be considered in the evaluation of any underwater acoustic classifier, since misclassifications are likely to result if the confounding effects of temperature, depth and boundary conditions are not taken into account by the system.

### 2.2. Sound Navigation and Ranging (SONAR) Systems

In general terms, a sonar system consists of a sensor, or an array of sensors (hydrophones, that are essentially transducers or *underwater microphones*), converting acoustic pressure underwater to electrical voltage [21].

Passive sonar systems detect acoustic signals emitted by objects of interest (ship’s propellers, seismic/volcanic or biological signals, for instance). The application of these systems assumes that all the information necessary for the detection, classification and tracking of underwater objects is available in the signal emitted by the objects themselves. Active sonar systems, on the other hand, have sound projectors that are transducers, converting electrical voltages to acoustic pressures. These projectors emit acoustic pulses (called *transmit waveform*), whose reflections are measured by the hydrophones. In these systems, the information necessary for achieving the inferential objectives reside in the reflection and scattering that the original signal suffers upon interacting with objects in the environment.

Any sonar application (involving machine-learning processing or not) should take into account the limitations of the sensing apparatus. Usually, this is measured by accounting for each part of the system, including the system’s components, the effects of the underwater environment, and the characteristics of sound or scattering from the target. This accountability is summarised in sonar Equations (3) and (4) [21], resulting in the Signal-to-Noise Ratio (SNR) of the system. For a source level (SL), a propagation loss (PL), a noise power level in the processing band (NL) and an array gain (AG) (all measured in decibels), the basic *passive* sonar equation is given by:

(3) $$\begin{array}{c} SNR_{Passive}=SL-PL-NL+AG. \end{array}$$

The basic *active* sonar Equation (4) also takes into account the target strength (TS), and the propagation losses from the sound projector ($PL_{a}$) and from the object of interest ($PL_{b}$), considering SL as the source level of the sound projector:

(4) $$\begin{array}{c} SNR_{Active}=SL-PL_{a}+TS-PL_{b}-NL+AG. \end{array}$$

While (3) and (4) somewhat incorporate the main underwater sonar sensing variables, including temperature, depth, salinity, air–sea and bottom–sea interfaces, rough boundaries and biological obstacles, most existing research on the application of machine-learning methods for the classification of underwater acoustic data (as we shall see below) ignores these effects, and does not evaluate the classification tasks with respect to the performance of the sonar system in distinct oceanographic situations.

Ignoring these variables limits the use of ML methods in applications that demand a high-level of trust, such as in defence systems or in the inspection of underwater built structures (such as deep-sea mining facilities). In these applications, classic acoustic processing strategies (not involving machine-learning algorithms) are still the first-choice methods because these effects can be explicitly accounted for.

A review of the literature on classic acoustic signal processing strategies for sonar data is outside the scope of this paper. However, as some machine-learning algorithms use these methods to preprocess the data, a few basic concepts pertaining to them are introduced below.

### 2.3. Main Concepts in Classic Acoustic Processing Methods

Acoustic signals can be understood as one-dimensional signals that oscillate in amplitude through time. Besides the many applications that can be developed using the temporal information of audio signals, some characteristics of this kind of data are best obtained in the frequency domain. In the particular case of underwater acoustic signal processing, the frequency domain is usually more informative [21]. To this end, Fourier transforms (FT) are used to obtain the frequency content of a time-domain signal [27]. The result of an FT on an autocorrelation function is called *power spectrum* that, informally, represents how much of the original signal is at a particular frequency.

Features related to amplitude and frequency can be directly analysed from the power spectrum, but the information of how the frequency varies with time is lost in this representation.

To fill this gap, two-dimensional time–frequency features can be extracted by passing a windowing function through the time signal and extracting the FT for each window of the original data. This procedure is called Short-Time Fourier Transform (STFT), and the unified resultant output is called the *spectrogram* of the signal. Spectrograms are typically plotted with time on the *x*-axis, frequency on the *y*-axis and magnitude on the *z*-axis. They can be thought of as a series of time-segmented FTs put in chronological order. The analysis of the harmonic behaviour of this spectrographic data is also an important feature for sound analysis in general [28,29,30], and can be obtained with the extraction of their *cepstrum* representation. The cepstrum is the result of the inverse FT of the signal spectrum logarithm, and is used to obtain the periodic structures in spectra [31]. The cepstrum is also commonly understood as the power spectrum of the logarithm of the power spectrum [21]. As the term cepstrum is a reversion of the first syllable of spectrum, operations on cepstra are also known as quefrency analysis (a semireversed spelling of frequency) [32].

Although acoustic signals are commonly represented as a one-dimensional continuous time series, humans do not perceive sound as a linear progression across frequency [33]. The Mel scale [34] is a logarithmic scale, emphasising low-frequency signals over high-frequency ones, and it is sometimes used for acoustic signal representation, aiming to mimic the human aural perception. On this scale, spectra represent the frequency features closer to the way sound is perceived by humans. Additionally, the frequency variations can be represented using the cepstrum of the Mel scale. The coefficients extracted from that cepstrum are called the Mel Frequency Cepstrum Coefficients (MFCC). The general process to extract MFCC from input signal is described in detail in [35]. Informally, MFCC is obtained by first sampling the original signal, then extracting the amplitude spectrum of each sample. After this process, the signal amplitude is converted to a logarithmic scale and also converted to the Mel scale. Finally, a discrete cosine transform (DCT) is taken to obtain the final form of MFCC.

Due to the fact that FT is composed of a sum of sinusoidal functions, this representation is not well-localised in time and space, since sine waves are functions with infinite duration. The wavelet transform was introduced to improve the representation of signals that have abrupt changes in time and space domains. A wavelet is a wave whose oscillation has a finite duration, which is defined in time, and has a zero mean [36]. Wavelets can be represented in many different formats, such as morlet, mexican hat, biorthogonal and others. Using the concepts of scaling, representing the frequency and duration of the wavelet, and shifting, representing the time positioning of the wave, this representation can capture both, high- and low-frequency features. The output of a wavelet transform is a matrix whose coefficients are functions of the scale and time information. Wavelet decomposition has proven to be suitable for analysing signals that contain information at different frequencies and time [37,38,39].

In the field of digital image processing, one common strategy used for texture analysis is the application of Gabor filters, which are linear filters that behave as a band pass, extracting frequencies in specific directions according to predefined kernels. There is some evidence that certain cells in the mammalian visual cortex (responsible for the perception of texture) can be approximated by such filters [40]. A common recent strategy in the definition of computational models of perception is to use Gabor filters as low-level visual primitives. Inspired by visual perception, some recent methods in acoustic processing use Gabor-filter banks on the sound spectra as a preprocessing step, achieving accurate results on acoustic processing tasks, such as the classification of environmental sounds [41], music genre recognition [42], and speech analysis [43,44], to cite a few.

When applied correctly, time–frequency analysis can yield useful insights into data. It does this by extracting and emphasising important signal characteristics, such as how frequency components (spectrogram) or harmonics of the frequency components (cepstogram) change over time. However, these approaches are nothing more than changing the way the data are represented and presented. They do not perform any sort of classification or categorisation of the information within the data. Traditionally, these higher-level pattern-matching tasks were performed by humans or basic linear or statistical models. However, over the last few years, machine learning in general, and neural networks in particular, have become the dominant way to classify patterns in noisy data. The following section will cover the basic understanding of deep neural networks.

### 2.4. An Informal Introduction to Deep Neural Networks

There have been significant recent developments in Deep Learning (DL) methods, mostly pushed by object detection and classification in images [45,46] and visual question answering [47]. Spectrographic (and cepstographic) representations of acoustic data are analogous to visual images in that they represent a signal across two dimensions (x, y spatial dimensions for images and time, frequency for spectrograms). Therefore, in order to introduce applications of DL in the classification of sonar data, a brief introduction to the application of these methods in image classification is in order.

The goal of object detection is to find instances of specific object classes in multiarray inputs, including colour images, videos, images from EM spectra or from other sources. Although this task is mainly investigated in the domain of computer vision [17], recent advances in Neural Network (NN) architectures, specialised in processing visual inputs (most notably Convolutional Neural Networks (CNN) [48]), have motivated the creation of new methods which have vastly improved the results of existing object detection and classification challenges [49]. Furthermore, many datasets of annotated images are readily available online, such as MS-COCO [50] and ImageNet [51], enabling the fast benchmarking of new methods. The open source availability of large, annotated image datasets has provided the appropriate basis for the rapid development of this field [52].

Informally, a CNN [53,54] extends the concept of an Artificial Neural Network (ANN) by adding a set of layers that function as *feature filters* (the convolutional layers), that, by enhancing specific aspects of the input, learn the features to be classified. More specifically, a CNN is usually structured in many stages. Initially, the input data are processed by a sequence of layers of two types: convolutional and pooling. The convolutional layers compute a sum of products, named *convolution*, with their input and a set of kernel weights, called *receptive fields*. This operation is applied at every spatial location of each element of the input data using a number of spatial increments called *strides*. A bias (or y-intercept) value is added to the result of this operation at each location, and the final value obtained is passed through an activation function. This is used to generate an input to the next layer as an array called *feature map*. Feature maps represent the extracted features from the original input (e.g., edges, points or blobs, considering images). Pooling layers are responsible for merging similar features by a subsampling process. Usually, the final stage of a CNN is a traditional, fully connected neural network, that learns class probabilities for the features picked out by the convolutional layers. The learning process of CNN occurs as an extension of back-propagation in fully connected neural networks, which propagates the gradient of an objective function through all the NN parameters (weights). This procedure is computed by gradient descent, for which various optimisation methods exist (such as Stochastic Gradient Descent (SGD), minibatch gradient descent method (MSGD), and Adaptive moment estimation (ADAM), amongst others [55]). A more complete, up-to-date introduction to DL and CNNs is described in [52].

Most deep-learning models for object recognition that exist today are variations of the basic CNN structure briefly explained above, with a distinct number, type and distribution of convolutional and pooling layers, or with some extra constraints on the way data are processed. There are three most-prominent CNN architectures that have a considerable influence on the image-classification area: AlexNet [56]; VGGnet [57]; and ResNet [58].

The AlexNet model [56] was proposed to solve the ImageNet Large Scale Visual Recognition Challenge (ILSVRC) [49], a competition for automatic object detection and classification based on the ImageNet [51] database. The model achieved an accuracy that was more than 10% better than the other state-of-the-art proposals in the competition. The original AlexNet architecture was composed of eight layers with learnable parameters, comprising five convolutional layers (with kernel sizes of 11 × 11, 5 × 5, 3 × 3, 3 × 3, 3 × 3), with layers one, two and five each followed by a Max Polling layer, and finally three successive fully connected layers. One of the major contributions of this model is the use of the Rectified Linear Units (ReLu) function as the activation layer. Before AlexNet, the most commonly used activation layers were based on *sigmoid* functions and *tanh* functions. ReLu offered a significant improvement in training time, as it is a simple max{0,x} function that presents a solution to the Vanishing Gradient (VG) problem [59]. As the Deep-Learning Networks go deeper (increasing the number of layers), there is a tendency for the value of gradients in the back-propagation algorithm to decrease. Due to the limits of *sigmoid* (0 to 1) and *tanh* (−1 to 1), the values of the derivatives become closer (but not equal) to zero, which causes the weight updates to be smaller as the error is propagated through the network, impacting the ability of the first layers to find appropriate weights in reasonable computational time. The ReLu function does not have the same limitation, since it has gradient of 1 when its input is greater than zero, and 0 otherwise, and so the back-propagation value does not diminish as it moves through the layers of the network.

To improve the convergence time of AlexNet, the VGGnet (or simply VGG) [57] was proposed to cope with the need to reduce the number of parameters necessary to obtain each feature map. The idea underlying this model is that the features that are usually learned with larger kernel sizes could be reproduced using a combination of smaller kernels (e.g., 3 × 3), reducing the number of parameters to be computed. This implies a reduced set of parameters, representing a faster execution and an improved robustness to overfitting. As we shall see later in this survey, VGGnets seem to be the model of choice in most work on the classification of objects from sonar data, as they present a good trade-off between classification accuracy and hyperparameter complexity.

Although the use of ReLu activation functions greatly improved the robustness of deep-learning models with respect to the VG problem, DL models were still difficult to train, and the accuracy of deeper models failed to perform better than shallower networks. ResNet [58] addressed this problem by introducing an *identity shortcut connection* between the convolutional layers. This identity shortcut connection is used to *skip* one or more layers, sending the output of previous layers to some layers ahead. This model is based on using a copy of the input data as base elements for the output values, forming a set named *residual blocks*. Residual blocks are composed of two sequential weight layers and one shortcut (identity layer) which connects the input directly to the output of the block. Sometimes, the weight layers, which can be convolutional, will not have the same dimensions, so a different type of shortcut is used, such as a convolutional with size reduction or even a padding applied on the input feature. The residual blocks aim to solve the *degradation problem*. This states that the accuracy function increases with the network depth only up to a certain number of layers, after which, adding more layers to the network causes the accuracy to decrease and the loss to increase relative to the shallower network. This is due to the complexity present in deeper models, which impacts the learning ability of the back-propagation algorithm. Identity shortcuts affect the updating of weights in the intermediate layers (those that are in parallel with the identity), generating an adaptability of the learning process only in cases where accuracy is improved. Thus, only the relevant layers (in terms of accuracy improvement) are considered in the final forward-pass of the trained network, and those layers that cause degradation generally converge to zero weights.

Another issue that any work in DL should consider is overfitting, which occurs when this model fits exactly with its training data, but does not generalise well to data outside the training set [60]. There are numerous strategies to tackle this issue, such as *early stopping* (stop the training before the model incorporates noise); *expand the training set* (more data implies a more accurate model); *feature selection* (this identifies the most relevant features to be learned, ignoring redundant ones); *ensemble methods* (that aggregate the output of a set of classifiers, selecting the best output by a voting process); and, finally, *regularisation* (that, in general, limits the amount of variance in the model by penalising input parameters with large coefficients). Regularisation methods have received great attention from recent studies [61], since they are solely related to the algorithms, and not to data quality or classifier competitions. One particular regularisation method worth mentioning in this review is *Dropout*, that ignores randomly chosen neurons (with a certain probability) during the training phase, so that a reduced network is obtained as a result. This strategy reduces the possibility for codependency among neurons, which is one factor that causes overfitting.

In the object-detection area, however, there are numerous architectures available that are also used for underwater acoustic signal processing. A useful method for grouping generic object-detection methods is to categorises them into two groups: *one-stage* detectors and *two-stage* detectors, as described below.

The *one-stage* detector category [62] includes algorithms such as *You Only Look Once (YOLO)* [63] and the *Single-Shot multi-box Detector (SSD)* [64], that use a single CNN to predict the bounding boxes locations, as well as the class probabilities of objects of interest. The original YOLO algorithm applies a single neural network to the input image. This network divides the image into a grid, while also generating bounding boxes using that grid as a base. Once the bounding boxes have been generated, class probabilities are assigned to each of them. In this way, YOLO considers the whole image at test time, making its predictions dependent of the global context depicted. In addition, the detection problem is treated as a regression, simplifying the pipeline, prioritising performance. More recently, new versions of the YOLO algorithm have been released (e.g., *Fast YOLO* [65], YOLOv2 [66], YOLOv4 [67] and PP-YOLO [68]) with increasing accuracy and processing speed.

Another neural network that fits into the one-stage detector category is the Single-Shot Detector (SDD) [64], which attempts to balance high accuracy and performance on object detection based on a feed-forward convolutional network. The model is based on the VGG convolutional neural network [57]. The SSD model extends VGG-16 with six additional convolutional layers, each producing feature maps at the scale of the targets. The additional layers defining an SSD model detect and classify large objects using feature maps of low resolutions, whereby high-resolution maps are used to detect and classify smaller objects.

In two-stage detectors, the system undertakes two distinct functions: the detection of regions of interests and the classification of objects in the regions of interest. The algorithm called *Regions with CNN Features* (R-CNN) [69] falls into this category, as well as its variation *Fast R-CNN* [70] that, instead of selecting regions from the input, generates regions from the convolutional feature maps. Fast R-CNN is faster than R-CNN since in the former the feature map is generated only once, rather than for every region selected (as imposed by the latter). The *Faster R-CNN* algorithm [71] eliminates the selective search method used in both R-CNN and Fast R-CNN, and uses a separate CNN (called *Region Proposal Network* (RPN)) to predict region proposals directly from the convolutional feature maps. The major contribution of these methods is the improvement of the accuracy and performance combined.

### 2.5. Neural Networks for Data Generation and Data Simplification

Two important classes of neural networks (that are largely outside the class of CNNs), Autoencoders (AEs) and Generative Adversarial Networks (GANs), are used to solve a variety of tasks related to the automatic classification of underwater acoustic data, as they are typically applied to generate data analogous to training data, rather than to classify objects of interest within the data.

In many instances, input data are a combination of an underlying pattern (signal) contaminated with noise. Noise is essentially uncorrelated with signal, thus with enough examples it is theoretically possible to abstract out desired patterns from noisy data. Autoencoders (AE) are an example of a NN model that executes this task [72,73]. Briefly, an AE attempts to replicate the components of the input that represent the common patterns, or signals of interest, while ignoring noise. In this way, AEs are more akin to a nonlinear denoising filter than a classifier since they do not attempt to categorise components within the input data [74]. Furthermore, AEs are an example of self-supervised learning (even though they are trained by means of supervised learning methods) since they do not need labelled input data to train from as they merely try to replicate a lower dimensionality/complexity version of the input. Since they can remove noise and simplify data, AEs can be used as a preprocessing step before a classical classifier (such as a SVM) [75] or even as components within a deep CNN [76,77].

Generative Adversarial Networks (GANs) [78] are two component deep neural networks that may or may not have convolutional layers. The first component, called *generator*, is trained to reproduce the patterns observed in the input data, *generating* as output synthetic data points that could have been part of the input (training) set. The other component, called *discriminator*, is trained to determine if the input is from the real dataset or synthetic data from the first GAN component. The two components making up the GANs architecture are trained simultaneously in a two-player adversarial zero-sum game. The training of a GAN stops when the discriminator classifies as real most of the synthetic examples generated by the generator, meaning that the first component is reliably creating data that are indistinguishable from the training set. Recently, GANs have been used as an important data-augmentation strategy, generating new datasets for training underwater acoustic object classifiers, as we shall see further in this paper.

### 2.6. Evaluation Metrics for Detection and Classification

Results of ML classification procedures in general (and detection in particular) can be summarised by a confusion matrix, which is a two-dimensional matrix representing in one of its dimensions (usually its *rows*) the correct instances of each class, whereas the other dimension (usually its *columns*) represents instances of the predicted class. In a binary classification task, with a distribution of *P* real positive instances and *N* real negative instances, the entries in a confusion matrix represent the number of *true positives* (TP) (correct predictions of the positive class, shown in its main diagonal); *true negatives* (TN) (correct predictions of the negative class); *false positives* (FP) (wrong predictions of the positive class); and, *false negatives* (FN) (wrong predictions of the negative class). The most usual metrics for evaluating a classification task are based on calculating rates from subsets of these values, such as *accuracy* ($\frac{TP+TN}{TP+TN+FN+FP}$), that measures the overall portion of correct classifications; *precision*, ($\frac{TP}{TP+FP}$) that is the fraction of *correct* positive instances identified by the classifier over all instances identified as positive for each class; *recall*, or *sensitivity* ($\frac{TP}{TP+FN}$), that measures the fraction of correct positive instances identified by the classifier over all positive instances for each class; among others [79,80]. The *F1 score* provides the weighted harmonic mean between precision and recall. Another important factor for evaluation purposes is the concept of *support*, which is the number of instances of each class in the test dataset (an imbalanced support has to be corrected, e.g., by sampling or rebalancing, in order to avoid bias in these metrics). These concepts can be extended to multiclass classification tasks by *microaveraging* or *macroaveraging*. The latter treats all classes equally by calculating the metrics for each class independently before taking the average, whereas the former averages over the combined contributions of all classes [80]. The area under the precision–recall curve for each class gives another important metric called *Average Precision* (AP), whereas the *Mean Average Precision* (mAP) score is obtained by taking the mean of AP over all classes.

These traditional metrics, however, propagate the underlying biases, ignore the trustworthiness of negative instances and do not take into account the chance-level performance [79,81]. Other common metrics that partially solve these issues include Cohen’s Kappa coefficient [82] and Mathews Correlation Coefficient (MCC) [83]. Cohen’s Kappa coefficient represents the degree of agreement between two predictors (or *raters*), calculated by means of the Accuracy (Acc) and the random accuracy (RA) (which is the agreement with a random classifier): $\frac{\left(Acc-RA\right)}{\left(1-RA\right)}$. The random accuracy can also be calculated using a confusion matrix:

$$RA=p_{1}p_{2}+\left(1-p_{1}\right)\left(1-p_{2}\right),\;\;where$$

$$p_{1}=\frac{TP+FN}{TP+FP+TN+FN}$$

and

$$p_{2}=\frac{TP+FP}{TP+FP+TN+FN}.$$

Cohen’s Kappa is an useful metric for imbalanced datasets; however, the interpretation of its value is problematic [84].

Mathews Correlation Coefficient (MCC) measures the differences between actual and predicted classes in a way that is analogous to $\chi^{2}$ statistics applied on a confusion matrix:

$$MCC=\frac{TN\cdot TP-FN\cdot FP}{\sqrt{\left(TP+FP)\cdot (TP+FN)\cdot (TN+FP)\cdot (TN+FN\right)}}.$$

MCC is a more reliable metric, whereby a high score is assigned only if the prediction was correct for the four base categories (TP, FP, TN and FP), taking into account the size of *P* and *N* [85]. We omitted from this overview the metric known as Area Under the Curve (AUC), which captures in a single value the Receiver Operating Characteristics (ROC) curve. Although AUC has been used to classify the accuracy of target detection algorithms [86], its extension to the analysis of multiclass classification is currently not totally understood [80,87].

Recently, Powers [79] argued that the notions of *informedness* and *markedness* overcome the issues related to most common metrics, such as accuracy, precision, recall and other related concepts. In its simplest form, informedness (or bookmarker informedness—BI) ($\frac{TP}{TP+FN}-\frac{FN}{TN+FP}$) measures how informed the rater is about true positives and true negatives (BI is recall with a correction for the negative instances); whereas markedness ($\frac{TP}{\left(TP+FP\right)}-\frac{FN}{TN+FN}$) measures how trustworthy the system’s predictions are (which is basically precision with a correction to account for the negative instances). The notion of area under the sample complexity curve (AUSCC) was recently introduced in [88] to provide an unbiased evaluation of ML models for the classification of underwater acoustic signals, taking into account the sample sizes per class. AUSCC is defined by the area under the curve, representing mean accuracy with respect to the number of samples in the training set.

Sokolova and Lapalme [80] present an analysis of 24 evaluation metrics for ML classification tasks, showing the existence of invariance properties across the metrics. These properties allowed the definition of a taxonomy matching the appropriate metrics with qualities of the datasets, such as representativeness of class distribution, reliability of class labels, and the unimodality or multimodality of the classes. These findings have yet to be applied for the evaluation of underwater acoustic automatic classification tasks, whose current literature (as we shall see later in this paper) assumes accuracy or mAP as the main evaluation metric.

## 3. Machine Learning for the Classification of Underwater Acoustic Signals

This section contains a review of the methods used for classification of underwater acoustic signals. These methods are separated into five main classes: methods that use convolutional neural networks alone; methods that apply biologically inspired feature-extraction filters as preprocessing; methods that use data from frequency and time–frequency analysis; methods using ML models as feature extraction layers; and classifiers based on transfer learning. Tables summarising these methods are presented below, relating the datasets used, the highest test result reported and a short description of the main contribution of each work.

### 3.1. Classification Using Only CNNs

Generally, in a time-series classification problem, the strategy is to first transform the data and represent it as a spectrogram or cepstrogram, then to obtain two-dimensional features by applying distance-based or feature-based methods [89]. The result of this process is used as input to a classifier. Although the application of this two-stage approach is not uncommon in this domain, the use of unprocessed audio signals that are directly filtered through convolutional kernels is also an option to tackle classification in the acoustic domain, as summarised in Table 1 and discussed below.

An end-to-end NN called “Auditory Perception-inspired Deep Convolutional Neural Network” (ADCNN) [90] was defined for underwater acoustic target recognition. It used a bank of multiscale deep convolutional filters as the first stage of the model to decompose the raw time domain signal into a set of distinct frequency domain signals. Each convolutional filter was followed by a max-pooling layer and several fully connected layers. After this, a fusion stage was defined in order to unify those components into a more informative representation (where the main distinguishing features were prominent). The data flow then goes through a decision layer, which produces the classification result. This process can be understood as an adaptive strategy where the subnetworks learn, directly from the input data, how to extract the intrinsic characteristics of the acoustic signal. The ADCNN model was able to execute each stage of the data-processing and classification pipeline, starting from noise decomposition, passing through feature extraction and finishing with the classification output. In contrast, the decision layer can also be replaced by other types of classifiers, such as *Extreme Learning Machine* (ELM) [96], which typically provides good generalisation performance when compared with densely connected neural networks for classification. Results presented in [91] show that the resulting CNN+ELM model was able to achieve a 93.04% recognition rate on a dataset of civil ships, representing an improvement of, at least, 6.79 percentage points when compared with other approaches that used classical features as inputs and fully connected layers as classifiers. The final analysis showed that the features generated by the one-dimensional convolutional layers acted as band-pass filters, similarly to the ADCNN model.

Similarly, a CNN model called Underwater Acoustic Target Classification DenseNet (UATC-DenseNet) [92] was designed to classify underwater acoustic targets from raw audio signals. This model is composed of a deep CNN with dense architecture that was trained to recognise 12 classes of signals. The dataset used was labelled by a sonar expert, containing 11 different classes of sounds and one class corresponding to the ambient sound. The impact on the final model accuracy was investigated by tuning the number of convolutional filters, the depth (number of layers) of the neural network model, the different layer configuration and the different input features. The results reported show that a particular model architecture (with six convolutional filters with a kernel size of 1 × 15), using the original sound data as input without preprocessing layers or filters, outperformed other classical methods, such as CNN-extreme learning machine [91], ResNet18 [58] and SqueezeNet [97]. The authors claimed that this performance improvement was due to the fact that the UATC-DenseNet optimised the use of features represented in multiple layers by an appropriate use of skip-connections (an argument that resembles the justification for the success of ResNet, cf. Section 2.4). Moreover, this work pointed to an important future direction that this type of research should take, which is the identification of topologies and parameters that are more appropriate for this kind of classification problem.

A multiscale residual deep neural network (MSRDN) (inspired by [94]) was developed in [93] to classify passive underwater acoustic signals by modelling the original signal waveform directly. The aim of using unprocessed original signal in this work was to avoid the loss of the waveform fine structure when converting it to the time–frequency domain and to reduce the dependency of the classification task on the window size of Fourier Transform (FT) and the hop length of the FT window. Tests using Ocean Networks Canada (https://data.oceannetworks.ca (accessed on 20 December 2021)) (ONC) public dataset have shown that MSRDN outperformed other DL methods whose inputs were based on time–frequency representations.

The gain in the automatic classification of raw audio signal relies on the possibility of extracting information without imposing any a priori hypothesis on the nature of the object to be identified and the medium in which it is immersed. This idea was explored two decades ago, and led to an early US patent [98]. In this work, a system that combines an unsupervised self-organised mapping (SOM) network [99] with learning vector quantisation (LVQ) [100] was proposed to provide high performance in classifying the roughness of the ocean floor from raw sonar data. This line of research, implemented in modern state-of-the-art hardware, which facilitates embedded parallel processing, could lead to deeper, more frequent instances of SOM networks and finer LVQs, possibly leading to more detailed representations of the seabed.

Virtually every efficient convolutional neural network in use today was first developed for image processing tasks. Therefore, most applications of CNNs to acoustic signals are based on adapting the methods initially developed for images. The development of dedicated acoustic feature-extraction strategies (using convolutional layers alone) to process audio signals is still an issue to be explored in this field.

### 3.2. Classification Using Biologically Inspired Feature Extraction Filters

Using as analogy the methods by which biological systems filter audio signals, a class of classifiers has evolved that uses bioinspired filters as a preprocessing step for detection and classification (Table 2).

Gabor-like spatial functions were applied as a bioinspired preprocessing stage in [62] for the automatic detection of minelike objects (MLOs) from side-scan sonar images. This method not only takes inspiration from the similarity of these functions with respect to early processing in the mammalian visual perception (cf. Section 2.3), but also from the fact that convolutional filters in CNNs often converge to Gabor functions [107]. The features extracted from these filters are commonly used as inputs for the deep network models [107,108]. In particular, a Gabor Neural Network (GNN) [107] uses Gabor filters at the first layers of a CNN, before any more generic convolutional layers, providing a set of initial features to be classified. The model was evaluated on a dataset of MLOs in sonar imagery provided by the Australian Defence Science and Technology Group (DSTG) in a naval mine-shape recovery operation in Australia. On that dataset, GNNs presented a better performance, with an average precision (AP) of 79.93%, than other state-of-the-art models, such as R-CNN, Fast R-CNN, Faster R-CNN, YOLOv3, Tiny YOLOv3 and SSD300, obtaining average precision metrics that varied from 9.41% to 72.76%. Furthermore, the proposed model presented a reduction factor of four with respect to the number of parameters needed for the other methods. As an end-to-end approach, the GNN represented a fast and reliable alternative to the other relevant MLO detection methods [109,110,111,112]. This was an indication that constraining certain network parameters, essentially prescribing their shape rather than using a generic structure, could be beneficial, since this reduces the number of parameters that need to be trained, hence reducing the amount of training data and training time required.

Ship-type classification from hydrophone data using CNNs was investigated in [101,102,103], where an algorithm was proposed that used a cochlea model to decompose the acoustic signal into target features. This was implemented as a convolutional layer with auditory filters that resemble the GNN [62]. The original (nonannotated) data used in this study is freely available at Ocean Networks Canada (https://www.oceannetworks.ca/ (accessed on 20 December 2021)) (ONC). The results showed that the proposed *auditory-inspired CNN* had a high accuracy (87.2%), outperforming a standard CNN (83.2%), a stack of autoencoders [113] (81.4%) and various traditional object recognition algorithms, such as a combination of wavelet transform with PCA [114] (74.6%) and a support-vector machine [115] (68.2%). However, Shen et al. [101] do not report the exact devices that were used, the precise period of data collection, or the labelled data used. Therefore, even though the original raw data are freely available, it is impossible to reproduce the results reported in that work.

The classification of large-scale acoustic signals was evaluated in [104], with a comparison among four popular CNN models (VGGnet, Inception, Xception, and DenseNet) and a classical baseline approach. The main aim of this comparison was to evaluate the improvement of using CNNs instead of classical acoustic classification methods. *Mel-filter bank features* were used to extract features from the signals. As the Mel scale emphasises lower frequencies over the higher ones (approximating the human auditory perceptions), using Mel-filter banks resulted in more lower frequency filters than filters on higher frequencies. This work showed that the logarithmic encoding of frequencies produced results 10 percentage points higher than linear encoding, which is further evidence that the use of preset preprocessing stages can result in improvements over random and generic starting points for a NN.

As CNNs can be understood as a combination of feature extraction layers (convolutions) and a classifier (the final densely connected layers), a few other recent contributions investigate the use of a single fully connected neural network to classify passive sonar data, receiving the extracted features from bioinspired methods as inputs. For instance, a fully connected neural network was used in [105] to classify data from two hydrophones, picking out signals from three distinct propellers running on a closed-water circuit cavitation tunnel. The raw data from the hydrophones were preprocessed into a feature vector formed by the discrete cosine transforms of the MFCC. The classifier used metaheuristic algorithms for optimising the weights and biases of the NN, aiming to avoid local minima, while accelerating learning convergence. The results presented were only compared with other metaheuristic algorithms training the same neural network, but not against state-of-the-art deep-learning methods. The data used are not available to reassess these results, making reproduction, or even comparison with the performance of modern classification systems, impossible. Nevertheless, the use of metaheuristics (usually inspired by insect behaviours) for optimising the NN training opens an avenue for investigating the application of these methods to finding the optimal structure of a NN, or for fitting the hyperparameters of more complex architectures [116].

Mel-frequency cepstral coefficients can be used as input to fully connected neural networks and directed to the problem of underwater acoustic recognition of ship types, marine mammals and underwater background noise with weak targets [106]. The method proposed in [106] used a combination of Gammatone Frequency Cepstral Coefficients (GFCC) and mode decomposition on the MFCC to extract features from the signal, that were then input to the neural network. Results obtained with this method suggest that by not using a CNN to learn the features saves computing time, as no redundant features are generated. However, comparisons were only executed against variations of the authors’ own algorithm, and not with respect to state-of-the-art CNNs.

Much of the work presented in this section was developed following a *problem solving* strategy, whose focus is on error minimisation under specific conditions, when the real effort should instead be employed on a *problem understanding* approach, where the background knowledge about the interrelation between environment conditions, SNR, and machine-learning tools dictates the future development of suitable combinations of filters with DL models. Nevertheless, in general, the use of bioinspired preprocessing steps in ML classification procedure has been shown to reduce the complexity of the ML training procedure, accelerating the learning process while avoiding local maxima.

### 3.3. Classifying Data from Frequency and Time–Frequency Analysis

Frequency and time–frequency analysis has been used to preprocess acoustic signals in order to enhance the most distinctive data features, facilitating the classification process. Table 3 presents a summary of important methods related to this idea, as described below.

Conventional algorithmic approaches to vessel classification and ranging from hydrophone data perform well at close range (<180 m) but their performance degrades quickly at longer ranges. These methods are also inaccurate in the presence of noise [121]. Using cepstrographic inputs to a CNN can overcome the shortcomings of the conventional approaches. The combination of cepstral filtering and CNNs greatly extends detection ranges and robustness to noise compared to an algorithmic method, but has slightly reduced accuracy at short range due to expressive bias [25]. Analogously, the performance of a standard CNN in the classification of surface vehicle propeller cavitation noise (VPCN) in shallow water has shown to be improved by analysing the amplitude variation of signal to detect the fundamental frequencies of VPCN [120]. Results described in [120] suggest that this method outperforms traditional classification methods. However, the fundamental frequencies of the target object need to be known beforehand, and it is assumed that they are independent of environmental conditions (which may not be true in the general case).

Ship classification from acoustic data is considered a binary classification problem in [6], aiming at identifying surfaces of underwater vessels. As the audio signals are received on an N-element vertical line array, each element of the original array is considered to generate two initial representations: the phone-space and the mode-space Cross-Spectral Density Matrices (pCSDM and mCSDM, respectively). A CSDM is a time–frequency representation used to compare different signals, similar to the covariance matrix, representing the distribution of power across the frequency spectrum over time. The absolute values of each CSDM element were used as the source for generating the two other matrices. Altogether, the generated matrices were used as input to several distinct machine-learning models: Random Forest, Support Vector Machine, a Feed-forward Neural Network and a CNN. All algorithms presented excellent performance, with an error rate below 5%. The training procedure employed in [6] used only simulated data considering a source with high SNR (2 dB to 5 dB), whereas the validation task was performed on a pair of simulated datasets: the first had an SNR of 2 dB to 5 dB; and the second had an SNR of −1 dB to 0 dB. The results reported confirm that the proposed machine-learning methods were robust with respect to SNR. However, it is unclear whether this result would generalise to real sonar data. Additionally, results suggest that phone-spaced data inputs are more suitable to ML methods than mode space, as the former are independent from environmental information.

In the need to improve the time–frequency representation of underwater acoustic signals, a method for sonar classification based on Anisotropic Chirplet Transforms (ACT) and deep learning was proposed [117]. Chirps are signals whose frequency increases or decreases with time. Similar to the relation of Waves to Wavelet (cf. Section 2.3), a chirplet is the time-limited representation of a chirp. They were first proposed to identify small iceberg fragments using sonar data [122], as those signals propagate on a radial chirp waveform. ACT was used in [117] as a preprocessing stage for an underwater acoustic communication dataset and for identifying whale sounds, then it was fed into a deep CNN, called time—frequency feature network (TFFNet). Additionally, an efficient feature pyramid (EFP) technique was defined by aggregating the context information of the features maps at different scales, widening the network instead of increasing its depth. This caused more features to be learned from the input data. The combination of ACT with the EFP method was compared against Random Forest and Support Vector Machines, achieving higher accuracy with less resources (memory and time). This result was attributed to the fact that ACT generated a high-resolution time–frequency representation, providing features that would not be achieved using other known transforms, such as STFT.

Discrete wavelet transform (DWT) was used in the classification of underwater acoustic signals with noise robustness in a method composed of four stages [118]: white noise elimination based on the DWT; an imaging stage, where the spectrogram of the discrete wavelet coefficients was obtained; a data-augmentation method (cf. Section 4.1); and the final classification stage. The data resulting from this four-tier process were then used as input to a standard CNN, composed of four convolutional layers, a dropout and a fully connected layer. The use of wavelet transform was shown to be effective at enhancing noise robustness in the system, achieving 99.7% classification accuracy with an SNR of 0 dB, greatly outperforming six other CNN architectures. The reason behind the improvement on noisy environments was attributed to the application of a threshold and also to the DWT coefficients on the noise elimination stage, impacting directly the performance of the wavelet denoising.

When using spectrograms of hydrophone data, a basic four-convolutional-layer CNN has been shown to outperform a fully connected NN with either 0 or 512-neuron hidden layers [119]. Furthermore, improved results were observed when both spectrograms and delta frequency images (i.e., the first difference of signal features, an approximation of the first derivative) were used as inputs over the use of spectrograms alone or in combination with delta–delta frequency images (i.e., the second difference of signal features). However, due to possible overfitting of the system resulting from small dataset size, high interclass similarity and a large number of model parameters, this finding may have limitations. This problem could be avoided using data-augmentation techniques (see Section 4 below).

A fully connected neural network was applied to recognise ships in hydrophone data [19]. In order to do this, the original signal was treated by traditional image-processing methods (mainly a short-time Fourier transform) to generate a binary image, based on the frequency spectrum of signal segmentation. This process was shown to improve the analysis of short-time transient sound behaviours, generating a better representation of the signal to be inputted into the binary image generation. An experiment with three different SNR (without noise; 5 dB and 10 dB) compared the accuracy of the proposed model with a fractal-based method, showing that NN have a better noise robustness for all analysed scenarios. Results also indicate that for the five-class classification problem, the feature extraction method proposed achieved a high-performance recognition with just 11 features.

Research has found that the classification of acoustic signals is improved when the data are presented in a time–frequency representation, such as a spectrogram or cepstrogram. Moreover, prefiltering in the time–frequency domain further improves classification as does the inclusion of convolutional layers within the classifier model.

### 3.4. Applying Machine-Learning Methods for Feature Extraction

This section discusses machine-learning methods used for extracting features from data, facilitating classification tasks. A summary of the methods overviewed in this section is presented in Table 4.

The scarcity of underwater acoustic data for training ML classification algorithms is a common statement in most related publications. In order to address this issue, Boltzmann Machine (RBM) autoencoders have been proposed in [123], aiming at reconstructing the original data to be used in the classification of vessels in passive sonar data. NN models that include RBM as a denoising element have been shown to greatly outperform the traditional Gaussian Mixture Model (GMM) with a Gammatone Frequency Cepstral Coefficient feature-extraction layer. In this way, the use of an unsupervised feature-extraction approach with RBM autoencoder (to provide noise extraction and signal reconstruction) and the combination of the power spectrum and demodulation spectrum into the input data (recognising important rhythm features of the original sound) have been shown to be a highly effective method for identifying ships from passive sonar signals.

The detection of underwater MLOs from synthetically generated data was attempted in [124], using a two-stage process. First, a one-class classification problem was solved by applying an autoencoder neural network to detect the target object (i.e., a mine and its shadow) without classifying the background; and second, the outputs of the autoencoders were used as inputs to a CNN that produced the class probabilities. In this context, detection occurs when the error between the input image snippet and the corresponding MLO generated by the autoencoder at the centre of that snippet is minimum. Although the results presented consider only computer-generated cylindrical mines, the use of autoencoders to extract mines before the application of a convolutional neural network reduced the amount of examples needed to train the latter. This process is akin to the *Region Proposal Network* implemented in the faster R-CNN [71] (described in Section 2.4). However, instead of searching for generic regions, autoencoders were used to identify specific shapes of MLOs within the input data.

Analogously to a Fourier transform, which can be represented as a convolution of the signal with the filter-bank impulse response, a set of convolutional filters was used in [125] as feature extractors to generate a time–frequency representation of the signals, which are compressed to the log scale to emphasise low frequencies. Then, the data served as input to two-dimensional convolutional layers, which acted as spectrotemporal feature learners, attached to a Bidirectional-LSTM layer with a trainable attention module, that was used to capture temporal relations. This system was tested on a dataset obtained in shallow waters in the Indian Ocean, achieving high-accuracy results. The analysis of the learned features showed that the convolutions applied were able to produce a behaviour equivalent to a Short-time Fourier transform, with adaptive parameters learned from the original raw data.

Machine learning is proving to be an important tool for underwater target classification where both unsupervised and self-supervised learning strategies (autoencoders) are applied to autonomously extract relevant features from acoustic data. Novel methods are successfully employing a combination of classic signal processing methods (such as STFT) with ML architectures [125] as a strategy to compensate the signal complexity by selecting its most distinctive features for the classification procedure. However, a challenging task still remains, that is, to find suitable hyperparameters for the DL architectures. Tools for automated machine learning [126] could provide an avenue to mitigate this issue. However, to the best of our knowledge, this is still a task for future work in this field.

### 3.5. Transfer Learning

A classic supervised learning task assumes the existence of a large amount of labelled data of the domain in which the learning will be applied. In most application domains, and in particular in the classification of sonar data, the labelled data available are not sufficient to train models that are reliable enough to be useful in situations that demand a high degree of accuracy, such as the automatic surveillance of shorelines. Transfer learning (TL) [127] is a machine-learning technique that solves this issue by leveraging the reduced amount of labelled data in a domain (target domain) by transferring and reusing the model learned on a distinct, but related, domain (source domain). In other words, TL methods extract the knowledge from one or more source tasks and apply it to a target task. TL has many advantages in the development of ML algorithms, such as shorter training times and the enhanced robustness of using models that have already passed initial tests. In particular, the ability of TL to produce good results with reduced datasets causes a positive impact on the classification of underwater acoustic data, since the acquisition and consequent availability of data is a constraining factor in general. This section discusses a few key contributions in this area, as summarised in Table 5.

TL has been used to create an automatic, multiclass object classifier using data from side-scan sonars for a range of applications, including the detection of sunken ship wrecks and aircraft, drowning victims, and MLOs [128]. A CNN model was created based on transfer learning, where a VGG-19 network was pretrained with ImageNet data and all of its trained layers. The last fully connected layers of this model were transferred to a new CNN, which was fine-tuned with a semisynthetic sonar dataset. The results reported in [128] were compared with more traditional methods for object classification, such as Support Vector Machines, a shallow CNN [134] and Deep Forests [135], where the TL-CNN showed better performance and accuracy. However, no comparison was described with respect to state-of-the-art methods for object classification from sonar, such as GNNs [62] (described at Section 3.2). Nevertheless, this work demonstrated that having small amounts of labelled training data need not preclude the creation of a useful and accurate classifier.

Following from the finding that TL is potentially beneficial, a set of pretrained neural networks for sonar images have been produced which are aimed at providing a basis for improving sonar classification results by means of transfer learning [88]. The aim was to accelerate the development of object classification for underwater acoustic data, using as analogy the positive momentum given to image classification with the introduction of ImageNet [51]. The models trained include various state-of-the-art convolutional and autoencoder architectures, that were trained on the Marine Debris Turntable (MDT) dataset (described in Section 4 below). The pretrained models are freely available for download (https://paperswithcode.com/paper/pre-trained-models-for-sonar-images (accessed on 20 December 2021)). The results presented support the authors’ initial hypothesis that transfer learning using pretrained models can be used successfully to obtain high accuracy in the classification of sonar data, using fewer samples to retrain the models. Moreover, although various machine-learning models were trained, there was no architecture that showed a superior performance over all training/test scenarios, which is a common issue found in machine learning, known informally as the “no free-lunch theorem” [136], where no one model is the best under all situations. The MDT dataset was used in [131] to evaluate the performance of transfer learning using the recognition accuracy as a function of the feature vector size, the object size as well as the training set size. Results show that CNNs (even when trained with distinct datasets from the target) are capable of learning relevant features, improving the accuracy of classification using transfer learning. Additionally, CNN models are largely invariant to the object size, and finally, a combination of ADAM optimiser with Dropout regularisation was shown to be a good strategy to cope with the small sample size problem.

Analogous to the work described in [88], a CNN was pretrained with images from the ImageNet dataset [51] and fine-tuned with a small sonar dataset for the classification of forward-looking sonar (FLS) data [129]. The main hypothesis underlying this work was to use the CNN to extract general features from optical images, that would enable accurate classification of sonar data by training (using transfer learning) using a small sonar dataset. The maximum accuracy achieved in this study was 97%, thus supporting the hypothesis that the feature-extraction process, obtained with pretrained CNN, was effective for classifying low-resolution FLS images.

A distinct policy for transfer learning for sonar image classification was followed in [132], where a convolutional neural network (VGG-19) was pretrained on the ImageNet dataset [51] and then fine-tuned to classify side-scan sonar data. This fine tuning was accomplished using a synthetic dataset obtained by transforming optical images into side-scan sonar outputs by means of a style-transfer algorithm (see Section 4). This work achieved an accuracy of over 97% in the classification of real sonar images for the CNNs that were fine-tuned with the synthetically generated dataset, which suggests that transfer learning (even using synthetic data to fine-tune the model) is the best strategy to take when large, annotated datasets are not available.

Instead of defining the entire end-to-end pipeline, two off-the-shelf CNN classifiers (pretrained in ImageNet dataset) were applied in [130]: AlexNet [56] and GoogLeNet [137], that were fine-tuned to identify human body shapes from sonar images. Two classes of datasets were used in this work: the first was obtained in a controlled environment and augmented synthetically (cf. Section 4.1); the second was obtained from real maritime situations off the west coast of Korea. The accuracy obtained testing the CNN with the real-world set of images revealed a poor performance of AlexNet overall, whereas GoogLeNet obtained an accuracy of 91.6% when trained with input data with various noise levels. This finding suggests that the GoogLeNet architecture, which is characterised by having convolutional filters of multiple sizes operating at the same level (therefore, making the network wider rather than deeper), is a suitable strategy for automated classification in this domain.

Side-scan sonar images were used as inputs to a real-time automatic target recognition (ATR) method that applied a combination of a transformer attention mechanism [138] and the YOLOv5s model [133], named TR–YOLOv5s. A complete pipeline was proposed, including preprocessing, sampling, target recognition and target localisation. Focusing on real-time operation, a two-fold strategy was pursed. Firstly, a preprocessing downsampling method was applied to the side-scan sonar input, maintaining the aspect ratio between the along-track direction and the cross-track direction. Secondly, the YOLOv5s architecture was used, aiming to take advantage of the fast detection speed and the high precision rate of the original model. Additionally, due to the sparse presence of targets on the seabed, the use of the transformer attention mechanism in the detector allowed the identification of areas of interest from the side-scan sonar images, focusing the operation of the model on the targets, and not on the background or blank areas. The YOLOv5s model reused the weights trained on the COCO dataset, improving the generalisation ability and reducing the final training time. Additionally, the application of the transformer helped to overcome the problem of the different target–background ratio between object detection datasets and side-scan sonar datasets.

Transfer learning is applied as an alternative to the scarcity of real-word data to train DL models, enabling the reuse of well-established results obtained from other domains. The reuse of trained architectures has shown positive results on the convergence and adaptability of two-dimensional convolutional filters, where features learned from colour images (such as the ones present on ImageNet dataset) are *transferred* to process 2D acoustic data. Unless some effort (and funding) is directed toward providing an underwater acoustic analogue of ImageNet, transfer learning, combined with data-augmentation methods discussed in the next section, is the next best valid option for developing useful DL models for sonar classification.

## 4. Datasets and Data-Augmentation Methods

CNNs have proved to be an important tool for automatic data classification. However, they are *data hungry*, as a large amount of labelled data points is needed to properly train and validate the models. This is a critical issue in the classification of underwater acoustic data, since most datasets are not publicly available, owing to the financial and technical complexity in obtaining such data and also to their potential defence-sensitive information. Therefore, much work in this area is conducted using synthetic data only [124], or on a limited set of real data augmented with semisynthetic examples for training [62,128,139]. There are, however, a few datasets commonly used in the literature, that are summarised in Table 6.

The next section describes data-augmentation methods, how they are commonly used for the classification of underwater acoustic data, and the methods available to evaluate their quality.

### 4.1. Data Augmentation and the Classification of Underwater Acoustic Data

This section presents some of the data-augmentation strategies used for training classifiers for underwater acoustic signals, as summarised in Table 7.

Data-augmentation methods aim to build synthetic data by transforming the existing labelled data using various transformations. Data-augmentation approaches can be based on time series, going from basic augmentations of time or frequency domains (such as cropping, slicing, and adding noise) to more advanced methods (such as Deep Generative Models, Statistical Generative Models, Decomposition-based, and others) [153]. The augmentations can be implemented either *online*, when the data are being augmented during the training stage, representing a new layer of the NN or a preprocessing stage during execution, which directly impacts the execution time; or *offline*, which is when the data are augmented before the execution of the training rounds, impacting directly on storage space.

One of the known problems with antisubmarine sonars is the number of false alarms, especially in nearshore regions due to the complex structure of sound propagation in these areas (cf. Section 2.1) [154]. An augmented dataset was introduced in [139] for training an ensemble of CNNs to classify false alarms from sonar data obtained in a nearshore environment. The base data used to this end was obtained from towing an active, lower frequency, sonar array in the Norwegian Trench close to the coast. In order to obtain a balanced set of examples to train the CNN model, since the number of false alarms is much greater that the number of true positives, each target detection was copied a number of times, while only a fraction of the false alarms were considered. The results presented show an unquestionable improvement in the classification of false alarms, in comparison with the usual method of thresholding a signal-to-noise ratio of the detections. It is worth pointing out that this augmented dataset was only used in the training phase, whereas the test set consisted only of original (nonaugmented) data points. Analogously, a data-augmentation procedure was performed in [118] by convolving an acoustic signal and an impulse response signal. Then, white Gaussian noise was added to finally produce the extended signals. After the data-augmentation process, a set containing 30,000 images of 30 different classes of underwater acoustic signals was generated. The results reported showed a reduction in overfitting and an improvement in generalisation ability with the use of the augmented dataset.

The *Deep Gabor Neural Network* [62] (cited in Section 3.2 above) also relied on a large number of training data to be tuned. In order to generate a dataset large enough for training the models, the original dataset was augmented by overlaying MLOs image snippets on images of various seabed backgrounds. To avoid invalid data generated from the augmentation, the overlaying process preserved the shadow direction of the MLO aligned to the shadow direction in the background image. However, there is uncertainty about the realism of the augmented dataset used. In a similar approach [128] (cited in Section 3.5), in order to generate semisynthetic images, a model extracts objects of interest (such as an aeroplane or a drowning victim) from the original image, superimposes these onto a randomly selected seabed background, and simulated shadows are then added according to the probability distribution of a real sonar reference image. The quality of the generated dataset was evaluated, in order to avoid bias, by means of two methods: one subjective, based on the evaluation of the images by human subjects; and one objective, where the Fréchet Inception Distance [155] was used to measure the statistical deviation of the semisynthetic samples in contrast with those obtained directly from the sonars. The results show that the proposed system outperformed traditional ML methods, suggesting that a performance improvement can be achieved when using semisynthetic data generation in the training phase.

Photorealistic sonar pictures generated from 3D models were used in [124] (described in Section 3.4). As the training phase proposed in this work was focused on extracting the characteristics of the objects of interest, the areas of the data points (images) generated for training were mostly occupied by MLOs, while the background (seabed) was composed of white noise, preventing the AE model from learning spurious features from seabed textures. In contrast, the test set represented realistic backgrounds (synthetic renderings of seabed), while MLOs occupied a very small part of each image. The main limitation of this method was the difficulty in representing the sonar echo, which was ignored on the experiments reported. The presence of sonar echo generated from the mine itself can mask the real shape of the MLO, but it had a minor influence on learning the shadow concept [124].

Synthetic datasets were used as inputs to the DL models, reproducing the shallow-water environment of the East Sea in South Korea [6]. Simulated data from a normal-mode propagation model (KRAKEN [156]—Ocean Acoustics library: https://oalib-acoustics.org/ (accessed on 20 December 2021)) was used, resulting in a set with a high signal-to-noise ratio compared to real-world data. There were two distinct datasets used in this experiment, one generated with a distributed SNR with values from 2 dB to 5 dB, and another with an SNR from −1 dB to 0 dB. The data generated were modelled by Monte-Carlo simulation considering a vertical line array (VLA), then reorganised into two cross-spectral density matrices (one defined on phone space and the other on mode space) that were used as inputs to four ML algorithms: random forest, SVM, feed-forward neural network (FNN) and CNN. The convolutional model produced the best accuracy results; however, it also showed high variability with respect to the input data representation (where a phone-space representation presented as a more suitable input type, as discussed in Section 3), whereas the FNN was not affected.

A data-augmentation technique was used in [123] to double the size of the dataset used to create the ShipsEar dataset (Section 3.4). In this augmentation procedure, the original data were divided into five categories, with 600 samples each, then an RBM autoencoder was applied to reconstruct the original signals. The main idea was to generate an output signal with the same overall characteristics of the input signals, but decoded from the high-level probability distribution generated on the encoder stage of the RBM. The method was tested with four different synthetic datasets: a sinusoidal function with noise; a higher frequency sinusoidal function with noise; a cosine function with noise; and power spectrum data of the real signal. The mean squared error (MSE) between the reconstruction and the original samples was in the range of 0.0115 to 0.5649, meaning that the difference between the two signals was minor and the properties were maintained. Therefore, using an autoencoder to extract the most relevant features from the signal and then in the decoder stage reconstructing the sound is a powerful strategy, as it can generate new signals with a representation derived from the same properties as the input data.

Similarly, an autoencoder based on a convolutional neural network was applied to perform sonar image noise reduction [146], generating a dataset of high-quality sonar images from a single image by applying the learnt autoencoder structures on the original sonar images for obtaining new examples.

There has been recent work on the application of Generative Adversarial Networks for generating new synthetic examples (trained to be similar to the items in a real acoustic dataset), in order to cope with the limited sample size in this domain. A typical GAN was applied to generate sonar images of MLOs [147], with which a combination of CNN and hierarchical Gaussian process classifier (CNN+HGP) was trained. The results show only a moderate improvement in the accuracy of the CNN+HGP classifier when using the augmented dataset (1.36 percentage points, achieving a maximum accuracy of 81.56%); a similar improvement was observed with respect to a stand-alone CNN (an improvement from 56.5% without the data augmentation to 57.2% with the GAN-generated dataset). Analogously, sonar data were generated using GAN in [148,149], where a simulator based on a ray-tracing method was used to generate simple synthetic sonar images containing shadows, background and foreground regions, and a GAN was used to make these simulated images similar to the data obtained from real sensors. GANs were also successfully used in [151] to produce high-resolution (long-array) sonar images from low-resolution (short-array) data. Sonar images were obtained by a GAN in [152] that accurately reproduced the data obtained by a moving vessel, creating analogous distortions due to the moving sensor. This idea can be used to train machine-learning systems both for vessel control and also for obtaining better images, given the vessel’s motion.

Side-scan sonar data were simulated in [132] using optical images *translated into sonarlike images* by a neural style-transfer (NST) algorithm [157]. NST is a technique that takes two images (called *content* and *style reference*) and blends them together, resulting in an output image that looks like the style reference. This is accomplished by optimising a loss function consisting of three parts: the content loss, which measures the distance between content and synthetic (generated) image by means of features extracted in the deep layers of a CNN; the style loss, which measures the distance between colour and texture (at various spatial scales) between the content and generated image; and finally, the total variation loss, which measures the spatial continuity between pixels of the synthetic image. The data were used to balance an uneven dataset, verifying that a model trained with synthetic data can improve both the classification accuracy and the convergence time.

A dataset for human body recognition from sonar images was proposed in [130], where the training portion of the dataset was obtained in a controlled environment, a 10 m-deep tank with a submerged dummy positioned 4 m below the surface. Additionally, the test dataset was obtained in the turbid waters of the west coast of Korea. A total of 31 base images were obtained in the controlled environment, that were augmented with various levels of background noise (salt and pepper) and polarising noise, resulting in datasets with 186 and 1860 images, respectively; whereas 30 images were obtained from the marine environment. The use of augmented data in the training stage proved to generalise well to the real-world problems, providing a classification accuracy of 91.6% using GoogLeNet as the classifier.

Data augmentation has proven itself to be a viable solution for the lack of annotated datasets, becoming an initial step for most of the studies related to the underwater acoustic domain. Self-supervised learning techniques, such as GANs and Autoencoders, are generally used for data generation, paving the way to more complex applications involving the combination of classical data-augmentation strategies with the ML approach. Although there has been an undeniable advance in this area, most approaches do not assess the characteristics of the data generated in comparison with the original ones, but measure the quality of the procedure through the accuracy of the classification, which represents a problem in the generalisation of such techniques.

## 5. Discussion and Concluding Remarks

A review of the literature related to the application of deep-learning methods for automatic object detection and classification of underwater acoustic data was presented in this paper. The literature discussed here mostly involves the classification of vessels from passive sonar data or the identification of objects of interest from active sonar. The present paper started with an informal introduction to underwater acoustics, which was then followed by a brief discussion of methods for acoustic data classification (including traditional tools as well as machine-learning-based methods), providing the background knowledge necessary to present the state of the art on the application of deep-learning methods to object detection and classification tasks from sonar data. This literature review (Section 3) was organised in five parts: (i) the application of deep learning (DL) methods using convolutional layers alone; (ii) DL methods that apply biologically inspired feature extraction filters as a preprocessing step; (iii) data classification from frequency and time–frequency analyses; (iv) methods using machine learning to extract features from original signals; and, (v) transfer learning methods. The chosen organisation of contributions is not intended to generate pairwise disjoint sets of works, as various references could be part of multiple classes (e.g., any method cited in (i)–(iv) could be used in a transfer learning setting (v)). Instead, this structure was used to make explicit some of the main strategies in machine learning that have been used in the classification of sonar data.

This paper also summarises some of the most important datasets cited in the literature, pointing out the notable scarcity of publicly available curated and annotated datasets (an issue that is often reported in research papers in this field [18]). Confidentiality issues, the cost and complexity of running real maritime missions, combined with the expensive task of curating, annotating and storing large amounts of data, are the main reasons for the nondisclosure of datasets. However, it also renders this type of research largely insubstantial, since without a common body of data, comparing and benchmarking solutions is a virtually impossible task. This lack of common datasets becomes even more evident when we take into account the complexity of the underwater domain (described in Section 2.1), where a number of factors influence the quality of data (such as time of day, season of the year, geographic regions, type of sensor, depth, pressure, etc.). To the best of our knowledge, there is no reference that evaluates the impact of these parameters with classifiers’ performance.

A variety of data-augmentation techniques have been used for coping with the scarcity of annotated datasets from real maritime missions (as described in Section 4). In fact, most of the literature overviewed in this paper uses some type of data-augmentation method to obtain a balanced dataset. Recent literature has described important positive results with the use of transfer learning (TL) techniques to tackle this issue as well (cf Section 3). However, it remains to be proven whether or not a distinct source domain (in TL), or the use of synthetic data, can fully represent the highly stochastic nature of the underwater acoustic domain.

The necessity to take a more scientific standpoint in the problem of automated classification of underwater acoustic data seems to be an imminent next step towards making progress in this field. Future work should consider making explicit the conditions of data acquisition and should also present a clear comparison of novel results against state-of-the-art methods using a common dataset. In order to accomplish such comparisons, both code and datasets used in the experiments should be made available along with their related publications. Otherwise, the very purpose of publishing results is defeated. Thus, it is of paramount importance that there is the creation of a set of freely available, curated, and annotated datasets for benchmarking, as well as the application of common appropriate evaluation procedures, possibly following the taxonomy proposed in [80]. This task could be easily accomplished with the creation of a sonar classification competition, similarly to the ImageNet Large Scale Visual Recognition Challenge (ILSVRC) (https://www.image-net.org/challenges/LSVRC/ (accessed on 20 December 2021)) that greatly motivated the fast development of artificial intelligence techniques to computer vision [49]. Associated with this, more investigation should also be conducted towards understanding and evaluating the existing state-of-the-art deep-learning architectures, and the various possible instantiations of their hyperparameters, applied to the classification of underwater acoustic signals. Additionally, the effect of the relative size of objects of interest with respect to background, the impact of training set size, the signal-to-noise ratio, as well as the consideration of environmental factors in the classification procedures, are important open issues that should be tackled in the future development of machine learning for underwater acoustic object classification.

The results summarised in this work show that accuracy is the most popular metric used for evaluating the underwater acoustic classification task, even though it is prone to produce biased results in certain situations (along with precision and recall [79]). Moreover, although accuracy is used for comparative analysis in the various publications in this area, these results cannot be generalised across distinct publications, since the test conditions (datasets, and underlying evaluation parameters) are unclear or unavailable. Therefore, it is virtually impossible to conclude on which methods are currently the most successful, or on promising future developments in this field. The metric values in Table 1, Table 2, Table 3, Table 4 and Table 5 were cited in this paper for completion purposes only, since the fundamental contribution of this review (given the current state of development of this field) is the description of the key recent ideas and the identification of some main pitfalls that future research should consider.

## Figures and Tables

**Table 1:** An overview of methods for classification using convolutional filters alone. In the ‘Metric’ column, ACCY stands for accuracy.

Reference	Network	Dataset	Metric	Main Contributions
Yang et al., 2019 [90]	Auditory Deep CNN	Ocean Network Canada signals ^a^	81.96% ACCY	The use of a bank of multiscale deep convolutional filters as a first processing stage, making possible the creation of an end-to-end NN.
Hu et al., 2018 [91]	CNN + ELM	Civil Ship ^b^	93.04% ACCY	The substitution of the final fully connected layer of a CNN for an ELM classifier, improving the generalisation of the model.
Doan et al., 2020 [92]	UATC-DenseNet	Real-world passive sonar data ^b^	98.85% ACCY	The analysis of the use of a number of convolutional blocks and layers, and different layer configurations and input features.
Tian et al., 2021 [93]	MSRDN	Ocean Network Canada signals ^a^	83.15% ACCY	The development of a deep residual network using the soft-thresholding proposed in [94] and the convolution kernel proposed in [95].

^a^ Dataset uses public data with nonpublic preprocessing techniques. ^b^ Dataset is proprietary and unavailable for reproduction.

**Table 2:** An overview of ML methods for classification using biologically inspired filtering algorithms. In the ‘Metric’ column, ACCY stands for accuracy, and AP stands for Average Precision.

Reference	Preprocessing	Network	Dataset	Metric	Main Contributions
Le et al., 2020 [62]	Gabor filter banks	Deep Gabor Neural Network	Australian DSTG data ^b^	79.93% AP	The use of Gabor filter to extract textures and outlines from the sonar images.
Shen et al. 2020, 2019, 2018 [101,102,103]	Cochlea model	Auditory Inspired CNN	Ocean Network Canada signals ^a^	87.2% ACCY	The use of Gabor filter layers inspired on the Cochlea model.
Wang et al., 2018 [104]	Mel-filter bank	Inception, Xception, VGG and Densenet	Whale FM	84.40% ACCY	The accuracy comparison of traditional methods and CNNs for the same scenarios.
Khishe and Mohammadi, 2019 [105]	MFCC	Fully Connected	Sonar dataset ^b^	97.12% ACCY	The accuracy comparison of metaheuristic algorithms and the use of fully connected NN.
Wang et al., 2019 [106]	GFCC and MFCC	Fully Connected	Six class dataset ^b^	94.3% ACCY	A combination of MFCC and GFCC was used as feature extraction showing time performance improvement.

^a^ Dataset uses public data with nonpublic preprocessing techniques. ^b^ Dataset is proprietary and unavailable for reproduction.

**Table 3:** An overview of ML methods for classification from frequency and time–frequency analysis. In the ‘Metric’ column, ACCY stands for accuracy, AP stands for Average Precision, and mAP stands for Mean Average Precision.

Reference	Preprocessing	Network	Dataset	Metric	Main Contributions
Ferguson et al., 2017 [25]	Cepstrum, Cepstrogram	CNN	Recorded boat radiated noise ^a^	99.78% AP	The cepstral representation of inputs for a NN and an improved distance estimation from acoustic noisy sources.
Choi et al., 2019 [6]	pCSDM, mCSDM	CNN, RF, SVM and FNN	Simulated acoustic data ^a^	>95% ACCY	Comparison between CNN and FNN.
Miao et al., 2021 [117]	ACT	TFFNet	Whale FM	92.1% mAP	The combination of ACT with the EFP to generate a high-resolution time–frequency representation.
Kim et al., 2021 [118]	DWT	CNN	Underwater acoustic signals ^a^	100% ACCY	The wavelet transform to obtain noise robustness and data augmentation as data source. The results converged with an 8-fold reduction in the number of epochs.
Cinelli et al., 2018 [119]	Spectrogram, delta, delta–delta frequencies	Fully Connected and CNN	Brazilian Marine dataset ^a^	88.1% AP	A comparative study of the accuracy of different ML architectures for various input layers.
Vahidpour et al., 2015 [19]	Image Histograms	Fully Connected	Five classes acoustic dataset ^a^	95.13% ACCY ^b^	The generation of a short-time Fourier transform-based binary image as input, improving noise robustness.
Bach et al., 2021 [120]	Signal demodulation	CNN	ShipsEar	90% ACCY	An algorithm for detecting the fundamental frequencies of a signal according to the amplitude variation, improving the performance of a CNN.

^a^ Dataset is proprietary and unavailable for reproduction. ^b^ Results obtained at 10 dB of SNR.

**Table 4:** An overview of methods for classification using machine learning for feature extraction. In the ‘Metric’ column, ACCY stands for accuracy.

Reference	Preprocessing	Network	Dataset	Metric	Main Contributions
Luo et al., 2021 [123]	RBM based autoencoder	BP Neural Network	ShipsEar	92.6% ACCY	Claims a better feature extraction performance than the conventional feature extraction methods.
Denos et al., 2017 [124]	Autoencoder NN	CNN	Synthetic realistic images ^a^	0.87 F1-Score	The combination of AE with CNN provided an application based on image object-detection models (R-CNN).
Kamal et al., 2021 [125]	Convolutional Filters	Deep convolutional LSTMs	Indian ocean acoustic dataset ^a^	95.2% ACCY	The proposal of an adaptive filter to generate time–frequency representation based on Short-Time Fourier Transform.

^a^ Dataset is proprietary and unavailable for reproduction.

**Table 5:** An overview of ML methods using transfer learning. In the ’Metric’ column, ACCY stands for Accuracy, and mAP stands for mean average precision.

Reference	Based Network	Dataset	Metric	Main Contributions
Huo et al., 2020 [128]	VGG-19	Seabed Objects-KLSG	97.76% ACCY	Fine tuning method with semi-synthetic dataset.
Fuchs et al., 2018 [129]	ResNet50, CNN-SVM	ARACAT dataset	90% ACCY	Results show that ImageNet can be used effectively by a CNN to extract general features that can then provide better accuracy when training a sonar classifier with small datasets.
Valdenegro-Toro et al., 2021 [88]	ResNet 20, MobileNets, DenseNet121, SqueezeNet, MiniXception, and Autoencoder	Marine Debris Turntable and Watertank, and Gemini 720i Panel-Pipe ^a^	96.31% ACCY	Reported results that reinforce the idea of achieving better accuracy with less data when using transfer learning.
Nguyen et al., 2019 [130]	AlexNet and GoogleNet	CKI, TDI-2017 and TDI-2018 ^a^	91.6% ACCY	The reuse of CNN architectures applied to image classification tasks.
Valdenegro-Toro, 2017 [131]	LeNet and SqueezeNet	Ocean Systems Lab water tank dataset ^a^	98.7% ACCY	Showed that transfer learning achieves acceptable accuracy even when the source and target sets have no classes in common.
Ge et al., 2021 [132]	VGG-19	Seabed Objects-KLSG and proprietary data ^a^	97.32% ACCY	Achieves high accuracy rate even with a small sample size obtained from a synthetically generated dataset.
Yu et al., 2021 [133]	Transformer and YOLOv5s	Two side-scan sonar datasets were constructed sonar images of shipwrecks and submerged containers.	85.6% mAP	The proposal of side-scan sonar automatic target recognition method, including preprocessing, sampling, target recognition and target localization, using the SOTA YOLOv5s model and an attention mechanism.

^a^ Dataset is proprietary and unavailable for reproduction.

**Table 6:** A summary of some datasets available.

Dataset	Description
Ocean Network Canada (ONC) [140]	A variety of (nonannotated) datasets containing continuously monitored data for relevant ocean variables on the east, west, and Arctic coasts of Canada are collected, maintained, and distributed by Ocean Networks Canada via their Oceans 3.0 data portal at https://data.oceannetworks.ca (accessed on 8 February 2022)
DeepShip: An Underwater Acoustic Benchmark Dataset [141]	DeepShip is a benchmark dataset (constructed with data from ONC) for underwater ship classification which consists of 47 h and 4 min of recordings of 265 different ships belonging to four different classes (background sound was not available). This dataset is available for download at https://github.com/irfankamboh/DeepShip (accessed on 20 December 2021)
ShipsEar: An underwater vessel noise database [142]	ShipsEar is a database containing underwater recordings of ship and boat sounds, which has 90 recordings of 11 different vessel types. It also has some useful information about the recordings, such as channel depth, wind, distance and location, to cite a few. This dataset is available for download at http://atlanttic.uvigo.es/underwaternoise/ (accessed on 20 December 2021)
Five-element acoustic dataset [143]	The main purpose of this dataset is to facilitate research on Doppler correction techniques for underwater acoustic transmissions. The dataset is composed of 360 communication packets with a duration of 0.5 s generated by a transceiver and captured by five hydrophones at nine different positions, and is available for download at http://users.ece.utexas.edu/~bevans/projects/underwater/datasets/ (accessed on 20 December 2021)
Fish classification with Dual-Frequency Identification Sonar (DIDSON) [144]	Fishery acoustic observation data were collected using Dual-Frequency Identification Sonar (DIDSON) with the purpose of classifying fish species. From 100 h of data, 524 clips were extracted with eight species labelled. The dataset is available for download at https://osf.io/sxek6/ (accessed on 20 December 2021)
Passive sonar spectrogram images derived from time series [145]	The main purpose of this dataset is to facilitate solutions for the problem of detecting tracks in a spectrogram. It contains 4142 spectrograms generated from synthetic and also real small-boat data. The dataset is available for download at https://sites.google.com/site/tomalampert/data-sets?authuser=0 (accessed on 20 December 2021)
Marine Debris Turntable (MDT) dataset [88]	This dataset was obtained from a forward-looking sonar (ARIS Explorer 300) placed in a water tank in which a rotating turntable was used to allow various poses for the objects observed. The MDT dataset contains 2471 images with 12 classes of object, including bottle, pipe, platform and propeller, and it is available from https://github.com/mvaldenegro/marine-debris-fls-datasets (accessed on 20 December 2021)

**Table 7:** An overview of the data-augmentation strategies.

Reference	Year	Data-Augmentation Method
Berg and Hjelmervik [139]	2018	Real target detections were copied multiple times and only a fraction of false alarms considered.
Le et al. [62]	2020	MLO image snippets overlaid on seabed background images.
Huo et al. [128]	2020	MLO image snippets overlaid on seabed background images with simulated shadows.
Denos et al. [124]	2017	Generated photo-realistic pictures from 3D mine object modelling combined with synthetically generated seabed background.
Choi et al. [6]	2019	Simulated data from a normal-mode propagation model, generated from Monte-Carlo simulation considering a vertical line array (VLA).
Luo et al. [123]	2021	Reconstruction of audio signals using the output from a RBM auto-encoder.
Kim et al. [146]	2017	Denoising of sonar images using the structures generated from an auto-encoder.
Kim et al. [118]	2021	Convoluted acoustic signals and impulse response signals, with white Gaussian noise added to generate extended audio signals.
Phung et al. [147]	2019	GAN generated sonar images of MLO.
Sung et al. [148]	2019	Ray-tracing method used to generate sonar images followed by a GAN to translate it into more realistic images.
Karjalainen et al. [149]	2019	Synthetic contact sonar images created using ray-tracing then refined using CycleGAN [150].
Rixon Fuchs et al. [151]	2019	Used a GAN on simulated data, to generate high resolution sonar data (long arrays) from low resolution counterparts (short arrays).
Jegorova et al. [152]	2020	Relied on the pix2pix architecture to create the Markov Conditional pix2pix GAN architecture, generating realistic-looking SSS images.
Ge et al. [132]	2021	Generated SSS data from optical images using a neural style-transfer.
Nguyen et al. [130]	2019	Added background noise (salt and pepper) and polarised noise to sonar images.

## Data Availability

No datasets were generated during this investigation.

## References

[1] Xu C., Chen J., Yan D., Ji J. (2016). Review of Underwater Cable Shape Detection. J. Atmos. Ocean. Technol..

[2] Chen X., Wu G., Hou S., Fan J., Dang J., Chen Z. (2019). Development of Tactile Imaging for Underwater Structural Damage Detection. Sensors.

[3] Orengo H., Garcia-Molsosa A. (2019). A brave new world for archaeological survey: Automated machine learning-based potsherd detection using high-resolution drone imagery. J. Archaeol. Sci..

[4] Dästner K., von Haßler zu Roseneckh-Köhler B., Opitz F., Rottmaier M., Schmid E. Machine Learning Techniques for Enhancing Maritime Surveillance Based on GMTI Radar and AIS. Proceedings of the 2018 19th International Radar Symposium (IRS).

[5] Terayama K., Shin K., Mizuno K., Tsuda K. (2019). Integration of sonar and optical camera images using deep neural network for fish monitoring. Aquac. Eng..

[6] Choi J., Choo Y., Lee K. (2019). Acoustic classification of surface and underwater vessels in the ocean using supervised machine learning. Sensors.

[7] Erbe C., Marley S.A., Schoeman R.P., Smith J.N., Trigg L.E., Embling C.B. (2019). The Effects of Ship Noise on Marine Mammals—A Review. Front. Mar. Sci..

[8] Merchant N.D., Pirotta E., Barton T.R., Thompson P.M. (2014). Monitoring ship noise to assess the impact of coastal developments on marine mammals. Mar. Pollut. Bull..

[9] Rossi E., Licitra G., Iacoponi A., Taburni D. (2016). Assessing the Underwater Ship Noise Levels in the North Tyrrhenian Sea. Adv. Exp. Med. Biol..

[10] Nastasi M., Fredianelli L., Bernardini M., Teti L., Fidecaro F., Licitra G. (2020). Parameters Affecting Noise Emitted by Ships Moving in Port Areas. Sustainability.

[11] McKenna M.F., Ross D., Wiggins S.M., Hildebrand J.A. (2012). Underwater radiated noise from modern commercial ships. J. Acoust. Soc. Am..

[12] Bocanegra J.A., Borelli D., Gaggero T., Rizzuto E., Schenone C. (2022). A novel approach to port noise characterization using an acoustic camera. Sci Total Environ..

[13] Childers D., Skinner D., Kemerait R. (1977). The cepstrum: A guide to processing. Proc. IEEE.

[14] Yang H., Lee K., Choo Y., Kim K. (2020). Underwater Acoustic Research Trends with Machine Learning: General Background. J. Ocean Eng. Technol..

[15] Yang H., Byun S., Lee K., Choo Y., Kim K. (2020). Underwater Acoustic Research Trends with Machine Learning: Active Sonar Applications. J. Ocean Eng. Technol..

[16] Yang H., Lee K., Choo Y., Kim K. (2020). Underwater Acoustic Research Trends with Machine Learning: Passive Sonar Applications. J. Ocean Eng. Technol..

[17] Khan A., Sohail A., Zahoora U., Qureshi A. (2020). A survey of the recent architectures of deep convolutional neural networks. Artif. Intell. Rev..

[18] Neupane D., Seok J. (2020). A Review on Deep Learning-Based Approaches for Automatic Sonar Target Recognition. Electronics.

[19] Vahidpour V., Rastegarnia A., Khalili A. (2015). An automated approach to passive sonar classification using binary image features. J. Mar. Sci. Appl..

[20] Kuperman W., Roux P. (2007). Underwater Acoustics. Springer Handbook of Acoustics.

[21] Abraham D.A. (2019). Underwater Acoustic Signal Processing Modeling, Detection, and Estimation.

[22] Urick R. (1979). Sound Propagation in the Sea.

[23] Leroy C.C., Robinson S.P., Goldsmith M.J. (2008). A new equation for the accurate calculation of sound speed in all oceans. J. Acoust. Soc. Am..

[24] McMahon K.G., Reilly-Raska L.K., Siegmann W.L., Lynch J.F., Duda T.F. (2012). Horizontal Lloyd mirror patterns from straight and curved nonlinear internal waves. J. Acoust. Soc. Am..

[25] Ferguson E.L., Ramakrishnan R., Williams S.B., Jin C.T. Convolutional neural networks for passive monitoring of a shallow water environment using a single sensor. Proceedings of the 2017 IEEE International Conference on Acoustics, Speech and Signal Processing (ICASSP).

[26] Weill A. (2014). Acoustic Waves, Scattering. Encyclopedia of Remote Sensing.

[27] Boashash B. (2016). Time-Frequency Signal Analysis and Processing.

[28] Tsai W.H., Lin H.P. (2010). Background music removal based on cepstrum transformation for popular singer identification. IEEE Trans. Audio Speech Lang. Process..

[29] Lee C.H., Shih J.L., Yu K.M., Lin H.S. (2009). Automatic music genre classification based on modulation spectral analysis of spectral and cepstral features. IEEE Trans. Multimed..

[30] Gopalan K. Robust watermarking of music signals by cepstrum modification. Proceedings of the 2005 IEEE International Symposium on Circuits and Systems.

[31] Oppenheim A.V., Schafer R.W. (2004). From frequency to quefrency: A history of the cepstrum. IEEE Signal Process. Mag..

[32] Bogert B.P., Healy M.J., Tukey J.W. The quefrency analysis of time series for echoes: Cepstrum, pseudo-autocovariance, cross-cepstrum and saphe cracking. Proceedings of the Symposium on Time Series Analysis.

[33] Oxenham A. (2018). How We Hear: The Perception and Neural Coding of Sound. Annu. Rev. Psychol..

[34] Stevens S.S., Volkmann J., Newman E.B. (1937). A scale for the measurement of the psychological magnitude pitch. J. Acoust. Soc. Am..

[35] Logan B. (2000). Mel frequency cepstral coefficients for music modeling. International Symposium on Music Information Retrieval.

[36] Donald D., Everingham Y., McKinna L., Coomans D. (2009). Feature Selection in the Wavelet Domain: Adaptive Wavelets.

[37] Tzanetakis G., Essl G., Cook P. (2001). Audio analysis using the discrete wavelet transform. Proceedings of the Acoustics and Music Theory Applications.

[38] Endelt L.O., la Cour-Harbo A. Wavelets for sparse representation of music. Proceedings of the Fourth International Conference on Web Delivering of Music, 2004. Edelmusic 2004.

[39] Chuan C.H., Vasana S., Asaithambi A. Using wavelets and gaussian mixture models for audio classification. Proceedings of the 2012 IEEE International Symposium on Multimedia.

[40] Jones J.P., Palmer L.A. (1987). An evaluation of the two-dimensional Gabor filter model of simple receptive fields in cat striate cortex. J. Neurophysiol..

[41] Souli S., Lachiri Z. Environmental sound classification using log-Gabor filter. Proceedings of the International Conference on Signal Processing Proceedings, ICSP.

[42] Costa Y., Oliveira L., Koerich A., Gouyon F. (2013). Music genre recognition using gabor filters and LPQ texture descriptors. Iberoamerican Congress on Pattern Recognition.

[43] Ezzat T., Bouvrie J.V., Poggio T.A. Spectro-temporal analysis of speech using 2-D Gabor filters. Proceedings of the Interspeech 8th Annual Conference of the International Speech Communication Association.

[44] He L., Lech M., Maddage N., Allen N. Stress and emotion recognition using log-Gabor filter analysis of speech spectrograms. Proceedings of the 2009 3rd International Conference on Affective Computing and Intelligent Interaction and Workshops.

[45] Liu X., Xu Q., Wang N. (2019). A survey on deep neural network-based image captioning. Vis. Comput..

[46] Hossain M.Z., Sohel F., Shiratuddin M.F., Laga H. (2019). A comprehensive survey of deep learning for image captioning. ACM Comput. Surv. (CsUR).

[47] Wu Q., Teney D., Wang P., Shen C., Dick A., van den Hengel A. (2017). Visual question answering: A survey of methods and datasets. Comput. Vis. Image Underst..

[48] LeCun Y., Bengio Y. (1998). Convolutional Networks for Images, Speech, and Time Series. The Handbook of Brain Theory and Neural Networks.

[49] Russakovsky O., Deng J., Su H., Krause J., Satheesh S., Ma S., Huang Z., Karpathy A., Khosla A., Bernstein M. (2015). ImageNet large scale visual recognition challenge. Int. J. Comput. Vis..

[50] Lin T.Y., Maire M., Belongie S., Hays J., Perona P., Ramanan D., Dollár P., Zitnick C.L. (2014). Microsoft coco: Common objects in context. European Conference on Computer Vision.

[51] Deng J., Dong W., Socher R., Li L.J., Li K., Fei-Fei L. ImageNet: A Large-Scale Hierarchical Image Database. Proceedings of the CVPR09.

[52] LeCun Y., Bengio Y., Hinton G. (2015). Deep learning. Nature.

[53] Rumelhart D.E., Hinton G.E., Williams R.J. (1986). Learning representations by back-propagating errors. Nature.

[54] LeCun Y., Boser B.E., Denker J.S., Henderson D., Howard R.E., Hubbard W.E., Jackel L.D. (1990). Handwritten digit recognition with a back-propagation network. Adv. Neural Inf. Process. Syst..

[55] Sun S., Cao Z., Zhu H., Zhao J. (2020). A Survey of Optimization Methods From a Machine Learning Perspective. IEEE Trans. Cybern..

[56] Krizhevsky A., Sutskever I., Hinton G.E. (2012). ImageNet classification with deep convolutional neural networks. Adv. Neural Inf. Process. Syst..

[57] Simonyan K., Zisserman A. (2014). Very deep convolutional networks for large-scale image recognition. arXiv.

[58] He K., Zhang X., Ren S., Sun J. Deep residual learning for image recognition. Proceedings of the IEEE Conference on Computer Vision and Pattern Recognition.

[59] Hochreiter S., Bengio Y., Frasconi P., Schmidhuber J., Kremer S.C., Kolen J.F. (2001). Gradient flow in recurrent nets: The difficulty of learning long-term dependencies. A Field Guide to Dynamical Recurrent Neural Networks.

[60] Brownlee J. (2018). Better Deep Learning: Train Faster, Reduce Overfitting, and Make Better Predictions.

[61] Tian Y., Zhang Y. (2022). A comprehensive survey on regularization strategies in machine learning. Inf. Fusion.

[62] Le H.T., Phung S.L., Chapple P.B., Bouzerdoum A., Ritz C.H. (2020). Deep gabor neural network for automatic detection of mine-like objects in sonar imagery. IEEE Access.

[63] Redmon J., Divvala S., Girshick R., Farhadi A. You only look once: Unified, real-time object detection. Proceedings of the IEEE Conference on Computer Vision and Pattern Recognition.

[64] Liu W., Anguelov D., Erhan D., Szegedy C., Reed S., Fu C.Y., Berg A. (2016). SSD: Single shot multibox detector. European Conference on Computer Vision.

[65] Shafiee M.J., Chywl B., Li F., Wong A. (2017). Fast YOLO: A Fast You Only Look Once System for Real-time Embedded Object Detection in Video. J. Comput. Vis. Imaging Syst..

[66] Redmon J., Farhadi A. YOLO9000: Better, faster, stronger. Proceedings of the IEEE Conference on Computer Vision and Pattern Recognition.

[67] Bochkovskiy A., Wang C.Y., Liao H.Y.M. (2020). Yolov4: Optimal speed and accuracy of object detection. arXiv.

[68] Long X., Deng K., Wang G., Zhang Y., Dang Q., Gao Y., Shen H., Ren J., Han S., Ding E. (2020). PP-YOLO: An effective and efficient implementation of object detector. arXiv.

[69] Girshick R., Donahue J., Darrell T., Malik J. Rich feature hierarchies for accurate object detection and semantic segmentation. Proceedings of the IEEE Conference on Computer Vision and Pattern Recognition.

[70] Girshick R. Fast R-CNN. Proceedings of the IEEE International Conference on Computer Vision.

[71] Ren S., He K., Girshick R., Sun J. (2016). Faster R-CNN: Towards real-time object detection with region proposal networks. IEEE Trans. Pattern Anal. Mach. Intell..

[72] Vincent P., Larochelle H., Lajoie I., Bengio Y., Manzagol P.A., Bottou L. (2010). Stacked denoising autoencoders: Learning useful representations in a deep network with a local denoising criterion. J. Mach. Learn. Res..

[73] Ng A. (2011). Sparse autoencoder. CS294A Lect. Notes.

[74] Wang W., Huang Y., Wang Y., Wang L. Generalized Autoencoder: A Neural Network Framework for Dimensionality Reduction. Proceedings of the 2014 IEEE Conference on Computer Vision and Pattern Recognition Workshops.

[75] Ju Y., Guo J., Liu S. A Deep Learning Method Combined Sparse Autoencoder with SVM. Proceedings of the 2015 International Conference on Cyber-Enabled Distributed Computing and Knowledge Discovery.

[76] Sun K., Zhang J., Zhang C., Hu J. (2017). Generalized extreme learning machine autoencoder and a new deep neural network. Neurocomputing.

[77] Huang F., Zhang J., Zhou C., Wang Y., Huang J., Zhu L. (2020). A deep learning algorithm using a fully connected sparse autoencoder neural network for landslide susceptibility prediction. Landslides.

[78] Goodfellow I., Pouget-Abadie J., Mirza M., Xu B., Warde-Farley D., Ozair S., Courville A., Bengio Y. (2020). Generative Adversarial Networks. Commun. ACM.

[79] Powers D. (2011). Evaluation: From Precision, Recall and F-Factor to ROC, Informedness, Markedness & Correlation. Int. J. Mach. Learn. Technol..

[80] Sokolova M., Lapalme G. (2009). A systematic analysis of performance measures for classification tasks. Inf. Process. Manag..

[81] Valverde-Albacete F.J., Peláez-Moreno C. (2014). 100% classification accuracy considered harmful: The normalized information transfer factor explains the accuracy paradox. PLoS ONE.

[82] Cohen J. (1960). A Coefficient of Agreement for Nominal Scales. Educ. Psychol. Meas..

[83] Matthews B.W. (1975). Comparison of the predicted and observed secondary structure of T4 phage lysozyme. Biochim. Biophys. Acta (BBA)-Protein Struct..

[84] Delgado R., Tibau X.A. (2019). Why Cohen’s Kappa should be avoided as performance measure in classification. PLoS ONE.

[85] Chicco D., Jurman G. (2020). The advantages of the Matthews correlation coefficient (MCC) over F1 score and accuracy in binary classification evaluation. BMC Genom..

[86] Uzair M., Brinkworth R.S., Finn A. (2021). Bio-Inspired Video Enhancement for Small Moving Target Detection. IEEE Trans. Image Process..

[87] Lachiche N., Flach P. (2003). Improving Accuracy and Cost of Two-Class and Multi-Class Probabilistic Classifiers Using ROC Curves. Proceedings of the ICML’03.

[88] Valdenegro-Toro M., Preciado-Grijalva A., Wehbe B. (2021). Pre-trained Models for Sonar Images. arXiv.

[89] Xing Z., Pei J., Keogh E. (2010). A brief survey on sequence classification. ACM Sigkdd Explor. Newsl..

[90] Yang H., Li J., Shen S., Xu G. (2019). A deep convolutional neural network inspired by auditory perception for underwater acoustic target recognition. Sensors.

[91] Hu G., Wang K., Peng Y., Qiu M., Shi J., Liu L. (2018). Deep learning methods for underwater target feature extraction and recognition. Comput. Intell. Neurosci..

[92] Doan V.S., Huynh-The T., Kim D.S. (2020). Underwater acoustic target classification based on dense convolutional neural network. IEEE Geosci. Remote Sens. Lett..

[93] Tian S., Chen D., Wang H., Liu J. (2021). Deep convolution stack for waveform in underwater acoustic target recognition. Sci. Rep..

[94] Zhao M., Zhong S., Fu X., Tang B., Pecht M. (2020). Deep Residual Shrinkage Networks for Fault Diagnosis. IEEE Trans. Ind. Informatics.

[95] Hannun A.Y., Rajpurkar P., Haghpanahi M., Tison G.H., Bourn C., Turakhia M.P., Ng A.Y. (2019). Cardiologist-level arrhythmia detection and classification in ambulatory electrocardiograms using a deep neural network. Nat. Med..

[96] Huang G.B., Zhu Q.Y., Siew C.K. (2006). Extreme learning machine: Theory and applications. Neurocomputing.

[97] Iandola F.N., Han S., Moskewicz M.W., Ashraf K., Dally W.J., Keutzer K. (2016). SqueezeNet: AlexNet-level accuracy with 50x fewer parameters and< 0.5 MB model size. arXiv.

[98] Chakraborty B., Kodagali V., Baracho J., Joseph A. (2002). System for Classifying Seafloor Roughness. U.S. Patent.

[99] Kohonen T. (1982). Self-organized formation of topologically correct feature maps. Biol. Cybern..

[100] Sato A., Yamada K., Touretzky D., Mozer M.C., Hasselmo M. (1996). Generalized Learning Vector Quantization. Advances in Neural Information Processing Systems.

[101] Shen S., Yang H., Yao X., Li J., Xu G., Sheng M. (2020). Ship type classification by convolutional neural networks with auditory-like mechanisms. Sensors.

[102] Shen S., Yang H., Li J. (2019). Improved Auditory Inspired Convolutional Neural Networks for Ship Type Classification. OCEANS 2019-Marseille.

[103] Shen S., Yang H., Li J., Xu G., Sheng M. (2018). Auditory Inspired Convolutional Neural Networks for Ship Type Classification with Raw Hydrophone Data. Entropy.

[104] Wang D., Zhang L., Lu Z., Xu K. Large-scale whale call classification using deep convolutional neural network architectures. Proceedings of the 2018 IEEE International Conference on Signal Processing, Communications and Computing (ICSPCC).

[105] Khishe M., Mohammadi H. (2019). Passive sonar target classification using multi-layer perceptron trained by salp swarm algorithm. Ocean Eng..

[106] Wang X., Liu A., Zhang Y., Xue F. (2019). Underwater Acoustic Target Recognition: A Combination of Multi-Dimensional Fusion Features and Modified Deep Neural Network. Remote Sens..

[107] Luan S., Chen C., Zhang B., Han J., Liu J. (2018). Gabor convolutional networks. IEEE Trans. Image Process..

[108] Zadeh M.M.T., Imani M., Majidi B. Fast facial emotion recognition using convolutional neural networks and Gabor filters. Proceedings of the 2019 5th Conference on Knowledge Based Engineering and Innovation (KBEI).

[109] Gebhardt D., Parikh K., Dzieciuch I., Walton M., Hoang N.A.V. (2017). Hunting for naval mines with deep neural networks. OCEANS 2017-Anchorage.

[110] Sawas J., Petillot Y. (2012). Cascade of boosted classifiers for automatic target recognition in synthetic aperture sonar imagery. Meet. Acoust..

[111] Barngrover C., Kastner R., Belongie S. (2014). Semisynthetic versus real-world sonar training data for the classification of mine-like objects. IEEE J. Ocean. Eng..

[112] McKay J., Gerg I., Monga V., Raj R.G. (2017). What’s mine is yours: Pretrained CNNs for limited training sonar ATR. OCEANS 2017-Anchorage.

[113] Cao X., Zhang X., Yu Y., Niu L. Deep learning-based recognition of underwater target. Proceedings of the 2016 IEEE International Conference on Digital Signal Processing (DSP).

[114] Shi M., Xu X. Underwater target recognition based on wavelet packet entropy and probabilistic neural network. Proceedings of the 2013 IEEE International Conference on Signal Processing, Communication and Computing (ICSPCC 2013).

[115] Yang H., Gan A., Chen H., Pan Y., Tang J., Li J. Underwater acoustic target recognition using SVM ensemble via weighted sample and feature selection. Proceedings of the 2016 13th International Bhurban Conference on Applied Sciences and Technology (IBCAST).

[116] Khishe M., Mosavi M., Moridi A. (2016). Classification of sonar target using hybrid particle swarm and gravitational search. Mar. Technol..

[117] Miao Y., Zakharov Y.V., Sun H., Li J., Wang J. (2021). Underwater Acoustic Signal Classification Based on Sparse Time–Frequency Representation and Deep Learning. IEEE J. Ocean. Eng..

[118] Kim K.I., Pak M.I., Chon B.P., Ri C.H. (2021). A method for underwater acoustic signal classification using convolutional neural network combined with discrete wavelet transform. Int. J. Wavelets Multiresolut. Inf. Process..

[119] Cinelli L., Chaves G., Lima M. Vessel Classification through Convolutional Neural Networks using Passive Sonar Spectrogram Images. Proceedings of the Simpósio Brasileiro de Telecomunicações e Processamento de Sinais (SBrT 2018).

[120] Bach N.H., Vu L.H., Nguyen V.D. (2021). Classification of Surface Vehicle Propeller Cavitation Noise Using Spectrogram Processing in Combination with Convolution Neural Network. Sensors.

[121] Ferguson B.G., Lo K.W., Thuraisingham R.A. (2005). Sensor position estimation and source ranging in a shallow water environment. IEEE J. Ocean. Eng..

[122] Mann S., Haykin S. (1991). The chirplet transform: A generalization of Gabor’s logon transform. Vision Interface.

[123] Luo X., Feng Y., Zhang M. (2021). An Underwater Acoustic Target Recognition Method Based on Combined Feature With Automatic Coding and Reconstruction. IEEE Access.

[124] Denos K., Ravaut M., Fagette A., Lim H.S. (2017). Deep learning applied to underwater mine warfare. OCEANS 2017-Aberdeen.

[125] Kamal S., Chandran C.S., Supriya M. (2021). Passive sonar automated target classifier for shallow waters using end-to-end learnable deep convolutional LSTMs. Eng. Sci. Technol. Int. J..

[126] Vanschoren F.H.K. (2019). Automated Machine Learning: Methods, Systems, Challenges.

[127] Pan S.J., Yang Q. (2009). A survey on transfer learning. IEEE Trans. Knowl. Data Eng..

[128] Huo G., Wu Z., Li J. (2020). Underwater object classification in sidescan sonar images using deep transfer learning and semisynthetic training data. IEEE Access.

[129] Fuchs L.R., Gällström A., Folkesson J. Object Recognition in Forward Looking Sonar Images using Transfer Learning. Proceedings of the 2018 IEEE/OES Autonomous Underwater Vehicle Workshop (AUV).

[130] Nguyen H.T., Lee E.H., Lee S. (2019). Study on the Classification Performance of Underwater Sonar Image Classification Based on Convolutional Neural Networks for Detecting a Submerged Human Body. Sensors.

[131] Valdenegro-Toro M. Best practices in convolutional networks for forward-looking sonar image recognition. Proceedings of the OCEANS 2017-Aberdeen.

[132] Ge Q., Ruan F., Qiao B., Zhang Q., Zuo X., Dang L. (2021). Side-Scan Sonar Image Classification Based on Style Transfer and Pre-Trained Convolutional Neural Networks. Electronics.

[133] Yu Y., Zhao J., Gong Q., Huang C., Zheng G., Ma J. (2021). Real-Time Underwater Maritime Object Detection in Side-Scan Sonar Images Based on Transformer-YOLOv5. Remote Sens..

[134] Luo X., Qin X., Wu Z., Yang F., Wang M., Shang J. (2019). Sediment classification of small-size seabed acoustic images using convolutional neural networks. IEEE Access.

[135] Zhou Z.H., Feng J. Deep forest: Towards an alternative to deep neural networks. Proceedings of the Twenty-Sixth International Joint Conference on Artificial Intelligence (IJCAI-17).

[136] Lattimore T., Hutter M., Dowe D.L. (2011). No Free Lunch versus Occam’s Razor in Supervised Learning. Proceedings of the Algorithmic Probability and Friends. Bayesian Prediction and Artificial Intelligence—Papers from the Ray Solomonoff 85th Memorial Conference.

[137] Szegedy C., Liu W., Jia Y., Sermanet P., Reed S., Anguelov D., Erhan D., Vanhoucke V., Rabinovich A. Going deeper with convolutions. Proceedings of the 2015 IEEE Conference on Computer Vision and Pattern Recognition (CVPR).

[138] Vaswani A., Shazeer N., Parmar N., Uszkoreit J., Jones L., Gomez A.N., Kaiser L.U., Polosukhin I., Guyon I., Luxburg U.V., Bengio S., Wallach H., Fergus R., Vishwanathan S., Garnett R. (2017). Attention is All you Need. Advances in Neural Information Processing Systems.

[139] Berg H., Hjelmervik K.T. Classification of anti-submarine warfare sonar targets using a deep neural network. Proceedings of the OCEANS 2018 MTS/IEEE Charleston.

[140] Heesemann M., Insua T.L., Scherwath M., Juniper S.K., Moran K. Ocean Networks Canada; Editing Status 2021-10-11; re3data.org—Registry of Research Data Repositories.

[141] Irfan M., Jiangbin Z., Ali S., Iqbal M., Masood Z., Hamid U. (2021). DeepShip: An Underwater Acoustic Benchmark Dataset and a Separable Convolution Based Autoencoder for Classification. Expert Syst. Appl..

[142] Santos-Domínguez D., Torres-Guijarro S., Cardenal-López A., Pena-Gimenez A. (2016). ShipsEar: An underwater vessel noise database. Appl. Acoust..

[143] Perrine K.A., Nieman K.F., Henderson T.L., Lent K.H., Brudner T.J., Evans B.L. (2009). University of Texas Applied Research Laboratory Nov. 2009 Five-Element Acoustic Underwater Dataset.

[144] McCann E., Li L., Pangle K., Johnson N., Eickholt J. (2018). An underwater observation dataset for fish classification and fishery assessment. Sci. Data.

[145] Lampert T.A., O’Keefe S.E. (2013). On the detection of tracks in spectrogram images. Pattern Recognit..

[146] Kim J., Song S., Yu S.C. Denoising auto-encoder based image enhancement for high resolution sonar image. Proceedings of the 2017 IEEE Underwater Technology (UT).

[147] Phung S.L., Nguyen T.N.A., Le H.T., Chapple P.B., Ritz C.H., Bouzerdoum A., Tran L.C. Mine-Like Object Sensing in Sonar Imagery with a Compact Deep Learning Architecture for Scarce Data. Proceedings of the 2019 Digital Image Computing: Techniques and Applications (DICTA).

[148] Sung M., Kim J., Kim J., Yu S.C. (2019). Realistic Sonar Image Simulation Using Generative Adversarial Network. IFAC-PapersOnLine.

[149] Karjalainen A.I., Mitchell R., Vazquez J. Training and Validation of Automatic Target Recognition Systems using Generative Adversarial Networks. Proceedings of the 2019 Sensor Signal Processing for Defence Conference (SSPD).

[150] Zhu J.Y., Park T., Isola P., Efros A.A. Unpaired image-to-image translation using cycle-consistent adversarial networks. Proceedings of the IEEE International Conference on Computer Vision.

[151] Rixon Fuchs L., Larsson C., Gällström A. Deep learning based technique for enhanced sonar imaging. Proceedings of the 2019 Underwater Acoustics Conference and Exhibition.

[152] Jegorova M., Karjalainen A.I., Vazquez J., Hospedales T. Full-Scale Continuous Synthetic Sonar Data Generation with Markov Conditional Generative Adversarial Networks. Proceedings of the 2020 IEEE International Conference on Robotics and Automation (ICRA).

[153] Wen Q., Sun L., Yang F., Song X., Gao J., Wang X., Xu H. (2020). Time series data augmentation for deep learning: A survey. arXiv.

[154] Thorpe S., Hall A. (1993). Nearshore side-scan sonar studies. J. Atmos. Ocean. Technol..

[155] Dowson D., Landau B. (1982). The Fréchet distance between multivariate normal distributions. J. Multivar. Anal..

[156] Jensen F.B., Kuperman W.A., Porter M.B., Schmidt H. (2011). Computational Ocean Acoustics.

[157] Gatys L., Ecker A., Bethge M. (2016). A Neural Algorithm of Artistic Style. J. Vis..

